# Hepatokine Pregnancy Zone Protein Governs the Diet‐Induced Thermogenesis Through Activating Brown Adipose Tissue

**DOI:** 10.1002/advs.202101991

**Published:** 2021-09-13

**Authors:** Jun Lin, Xiaoxiao Jiang, Meng Dong, Xiaomeng Liu, Qiwei Shen, Yuanyuan Huang, Hanlin Zhang, Rongcai Ye, Huiqiao Zhou, Chunlong Yan, Shouli Yuan, Xiangnan Wu, Li Chen, Yanfang Wang, Min He, Yi Tao, Zhaoyun Zhang, Wanzhu Jin

**Affiliations:** ^1^ Key Laboratory of Animal Ecology and Conservation Biology Institute of Zoology Chinese Academy of Sciences Beijing 100101 China; ^2^ University of Chinese Academy of Sciences Beijing 100049 China; ^3^ Institute of Neuroscience and Translational Medicine College of Life Science and Agronomy Zhoukou Normal University Zhoukou 466000 China; ^4^ Department of General Surgery Huashan Hospital Fudan University Shanghai China; ^5^ College of Agriculture Yanbian University Yanji 133000 China; ^6^ State Key Laboratory of Animal Nutrition Institute of Animal Science Chinese Academy of Agricultural Sciences Beijing 100193 China; ^7^ Division of Endocrinology and Metabolism Huashan Hospital Fudan University Shanghai China

**Keywords:** brown adipose tissue, hepatokine, intermittent fasting, obesity

## Abstract

Intermittent fasting (IF), as a dietary intervention for weight loss, takes effects primarily through increasing energy expenditure. However, whether inter‐organ systems play a key role in IF remains unclear. Here, a novel hepatokine, pregnancy zone protein (PZP) is identified, which has significant induction during the refeeding stage of IF. Further, loss of function studies and protein therapeutic experiment in mice revealed that PZP promotes diet‐induced thermogenesis through activating brown adipose tissue (BAT). Mechanistically, circulating PZP can bind to cell surface glucose‐regulated protein of 78 kDa (GRP78) to promote uncoupling protein 1 (UCP1) expression via a p38 MAPK‐ATF2 signaling pathway in BAT. These studies illuminate a systemic regulation in which the IF promotes BAT thermogenesis through the endocrinal system and provide a novel potential target for treating obesity and related disorders.

## Graphical abstract

This paper reports a novel hepatokine, pregnancy zone protein (PZP), which is significant induction by refeeding. Loss of function studies and protein therapeutic experiment in mice reveal that PZP promotes diet‐induced thermogenesis through activating brown adipose tissue (BAT) via p38 MAPK‐ATF2‐UCP1 signaling pathway in BAT. These studies provide a novel potential target for treating obesity and related disorders.

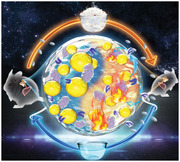

## 1 Introduction

The persistent imbalance between energy intake and energy consumption results in obesity, a major global health issue that continuously troubles the whole world with high risk occurrence of metabolic disorders, such as non‐alcoholic fatty liver disease (NAFLD) and type 2 diabetes mellitus (T2DM).^[^ ^1^ ^]^ Excessive energy storage as fat leads to the expansion of white adipose tissue (WAT). As a consequence, many metabolic disorders result from this increase in adipocyte size (hypertrophy).^[^ ^2^ ^]^

In contrast with WAT, brown adipose tissue (BAT), which owns multi‐ocular lipid droplets and numerous mitochondria, can uncouple substrate oxidation from ATP production into heat by utilizing uncoupling protein 1 (UCP1).^[^ ^3^ ^]^ Distribution of BAT was discovered by PET‐CT in adult human, and the reverse correlation of BAT activity with body mass index (BMI) make the process of BAT activation become the promising anti‐obesity therapeutic target.^[^ ^4^, ^5^ ^]^

As a safe and effective method for weight loss,^[^ ^6^, ^7^ ^]^ intermittent fasting (IF) regimen has different patterns according to different fasting and feeding windows, usually simplified as one day eating and one day fasting. It gained increasing attentions due to easier handling and remarkable ability to sustain weight loss.^[^ ^8^ ^]^ Several researches have proved that IF could elevate energy expenditure to reduce weight^[^ ^9^, ^10^, ^11^ ^]^ However, their molecular mechanisms involved in IF mainly focused on the adipose tissue itself, and it is still unclear whether there are inter‐organ communications playing a role in IF.

The majority of the regulatory responses to diet initially occur in the liver, and liver plays a key role in maintaining nutrient homeostasis by regulating the metabolism of other tissues,^[^ ^12^, ^13^ ^]^ wherein hepatokines act as the signal messengers. FGF21 was found to participate in the regulation of UCP1 expression of adipose tissue to promote energy expenditure.^[^ ^14^ ^]^ Apolipoprotein J (ApoJ), as a novel hepatokine, could regulate muscle glucose metabolism and insulin sensitivity through low‐density lipoprotein receptor‐related protein‐2 (LRP2)‐dependent mechanism.^[^ ^15^ ^]^ Considering the high sensitivity of the liver to metabolic changes during fasting and refeeding, we propose that liver secretory factors can promote energy expenditure, which contributes to the benefits of IF.

To identify the novel hepatokines that may play a role in IF, we performed a web‐based bioinformatic analysis and screened a protein, pregnancy zone protein (PZP), as a regulatory signaling molecule involved in IF. PZP, a member of *α*‐macroglobulin family,^[^ ^16^ ^]^ was originally known as a protease inhibitor because of its particular domain, a bait region that contains multiple protease cleavage sites.^[^ ^17^ ^]^ A report indicates that SNPs in PZP were associated with serum levels of alanine aminotransferase in NAFLD patients.^[^ ^18^ ^]^ Meta‐analyses of transcriptomics data from NAFLD patient livers have identified the expression of PZP was significantly downregulated in fatty livers compared with normal livers.^[^ ^19^ ^]^ Another study revealed that PZP level was higher in the weight‐loss group compared to the weight‐regain group.^[^ ^20^ ^]^ Although sparse reports have illustrated the involvement of PZP in metabolic diseases, the physiological role of PZP is not fully investigated. Our data illustrate a previously undefined role for refed‐induced PZP in systemic metabolic balance.

## 2 Results

### 2.1 Identification of PZP as a Hepatokine in Response to Refeeding

**Figure 1:**
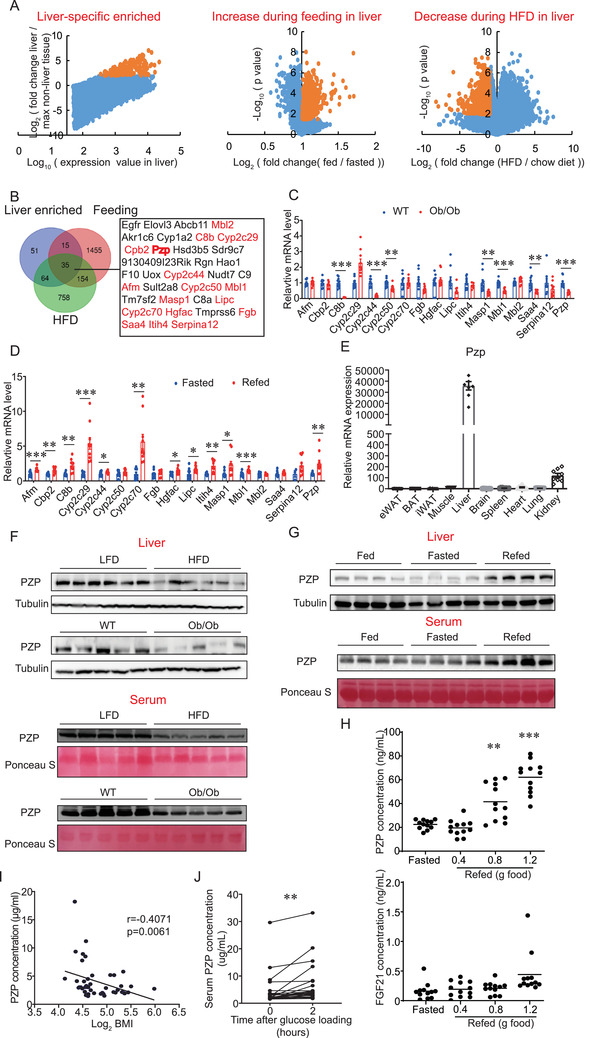
Identification of PZP as a hepatokine which is upregulated during refeeding. A) Scatter diagram of transcriptomic data‐mining strategy showing the three cohort of genes. Yellow dots indicate a cluster of genes which meet our determinative criteria. B) Venn diagram showing the overlap of three screened list. Gene name in red letter indicates a secreted protein. C) Relative mRNA expression of candidates in liver from WT or Ob/Ob mice (*n* = 7–10). 7 of total 17 detected genes were decreased in Ob/Ob mice compared with WT mice. D) Relative mRNA expression of candidates in liver from Fasted (24 h) or refed (6 h) mice (*n* = 9–10). 10 of total 17 detected genes were increased under refed condition. E) qPCR analysis of PZP expression in mouse tissues (*n* = 6–8). PZP was specific high expressed in liver than other tissue. F) Western blot analysis of PZP protein level in liver and serum from HFD mice or Ob/Ob mice which showed PZP protein levels were significantly down‐regulated in obese mice. G) Western blot analysis of PZP protein level in liver and serum after feeding or fasting (24 h) or refeeding (6 h) are shown. H) Serum concentration of PZP and FGF21 in mice after fasting (24 h) or different refeeding food intake (0.4, 0.8, 1.2 gram) which showed PZP expression were regulated by food intake in a dose‐dependent manner. I) Spearman correlation between circulating PZP concentration and log_2_ BMI which showed circulating PZP level inversely correlated with log_2_ (BMI). *χ* ^2^ tests were used for statistical analysis (*n* = 44). J) Serum concentration of PZP was significantly increased in human participants during OGTT. Paired sample *T*‐test was used for statistical analysis (*n* = 24). Data are shown as mean ± SEM. Two‐tailed Student's *t*‐test (C,D) or one‐way ANOVA (H) with multiple comparisons and Tukey's post‐test were performed; *** *p* <0.001, ** *p* < 0.01, and * *p* < 0.05 were considered to be significant.

A web‐based mice Affymetrix microarray data mining from NCBI database was performed to identify the potential hepatokines involved in refeeding induced thermogenesis and metabolic remodeling. In details, the gene expression profiles that satisfied all of the following criteria will be screened as the potential candidates. 1) The gene expression in the liver is abundant and at least twice as much as the highest expression level among all 21 non‐liver tissues. A total of 193 transcripts were screened out of 45 101 transcripts (NCBI GEO: GSE9954) (**Figure** **1**A, left); 2) The gene expression in the liver is significantly higher during feeding than that during fasting, 1851 transcripts were screened to meet these criteria (NCBI GEO: GSE46495) (Figure 1A, middle); 3) Expressions of many hepatokines were altered in the steatotic state. Most upregulated hepatokines cause metabolic dysfunction (e.g., Fetuin A,^[^ ^21^ ^]^ Fetuin B,^[^ ^22^ ^]^ Hepassocin,^[^ ^23^ ^]^ RBP4,^[^ ^24^ ^]^ Selenoprotein P^[^ ^25^ ^]^) and the rest of them suppress the aggravation of the disorder as a compensatory pathway (e.g., FGF21,^[^ ^14^ ^]^ ApoJ^[^ ^15^ ^]^). Unlike the complicated function of upregulated hepatokines, the downregulated hepatokines (e.g., Adropin,^[^ ^26^ ^]^ ANGPTL4,^[^ ^27^ ^]^ SHBG^[^ ^28^ ^]^) may be better targets which have positive metabolic actions. Therefore, we screened out the gene whose expression in the liver of mice with hepatic steatosis is significantly lower than that in healthy mice and 1191 transcripts were screened (NCBI GEO: GSE35961) (Figure 1A, right). We then merged these differentially expressed genes from three groups and finally screened 35 candidates that satisfied all three criteria (Figure 1B). Next, we identified 17 genes encoded secreted protein (indicated by red letters in the candidates list). We also confirmed these gene expressions in our samples using quantitative PCR (qPCR). As we observed, only expressions of three genes (C8b, Cyp2c44, and PZP) significantly decreased in the liver from obese mice (Figure 1C and Figure S1A, Supporting Information) and increased during refeeding compared to those from fasting state (Figure 1D). C8b encodes one of the three subunits of the complement component 8 (C8) protein, which is one component of the membrane attack complex and mediates cell lysis.^[^ ^29^ ^]^ Cyp2c44, belongs to the cytochrome P450 family, encodes cytochrome P450 epoxygenase.^[^ ^30^ ^]^ Hence, PZP was determined as the target of our following study.

Consistent with microarray data, our qPCR analysis confirmed the highest mRNA expression of PZP in mouse liver compared with other tissues (Figure 1E). We also confirmed the significantly decreased PZP concentration in the liver and serum from obese mice (Figure 1F and Figure S1B, Supporting Information). More importantly, both hepatic expression and serum level of PZP increased during refeeding compared to those from feeding and fasting state (Figure 1G and Figure S1B, Supporting Information). Refeeding‐induced expression of PZP only occurred in the liver but not in other tissues such as adipose, muscle and kidney (Figure S1C, Supporting Information). To reveal the effects of refeeding intensity on PZP expression, we measured the serum PZP levels of mice with different food intake within 6 h after 24‐h fasting. We found that the high refeeding intensity (0.8 and 1.2 g food) significantly induced high serum PZP levels, while the serum FGF21 was kept at a low level (Figure 1H). To investigate whether hepatocytes directly contribute to refeeding‐induced increase expression of PZP, we isolated the hepatocytes and detected the protein level of PZP in different culture conditions. We confirmed higher PZP expression in liver than that in BAT and its expression responded to the change of nutrients (Figure S1D, Supporting Information). These results suggest that diet‐induced PZP expression may play a specific role in metabolic remodeling. Glucose and insulin may be the potential factors that trigger the expression and release of PZP in this inducing progress.

To investigate whether the changes in PZP level also occurred in human obesity, we detected the serum levels of PZP in a cohort of individuals with a wide range of BMI and found that circulating PZP level inversely correlated with log_2_ (BMI) (Figure 1I). The clinical parameters were shown in Table S3, Supporting Information. To investigate whether refeeding induces PZP expression in human samples, the serum samples were collected from volunteers during an oral glucose tolerance test (OGTT). Interestingly, we found that 120 min after glucose loading, PZP secretion was significantly increased (Figure 1J). The clinical parameters were shown in Table S4, Supporting Information. Taken together, these data point to a potential physiologic function of PZP in metabolic remodeling and are suggestive of a link between PZP and refeeding.

### 2.2 Anti‐Obesity Effect of IF is Impaired in PZP Null Mice

To determine the physiologic role of PZP in energy metabolism, we generated the PZP global knockout (KO) mice. We found that PZP protein was depleted both in liver and serum from PZP KO mice (Figure S1E, Supporting Information). In other tissues (kidney, BAT, WAT, and muscle), PZP level was negligible both in WT and KO mice compared with that in liver from WT mice (Figure S1F, Supporting Information). PZP KO mice appeared normal by gross examination, and no significant changes in body weight, glucose tolerance and tissue weight were observed when fed chow diet compared with wild type (WT) mice (Figure S1G–I, Supporting Information). Upon HFD condition, PZP KO mice gained similar body weight to controls, accompanied by similar glucose tolerance, tissue weight, oxygen consumption, physical activity and food intake (Figure S2A–C,E–G, Supporting Information). Fatty liver was both observed in WT and PZP KO mice (Figure S2D, Supporting Information).

**Figure 2:**
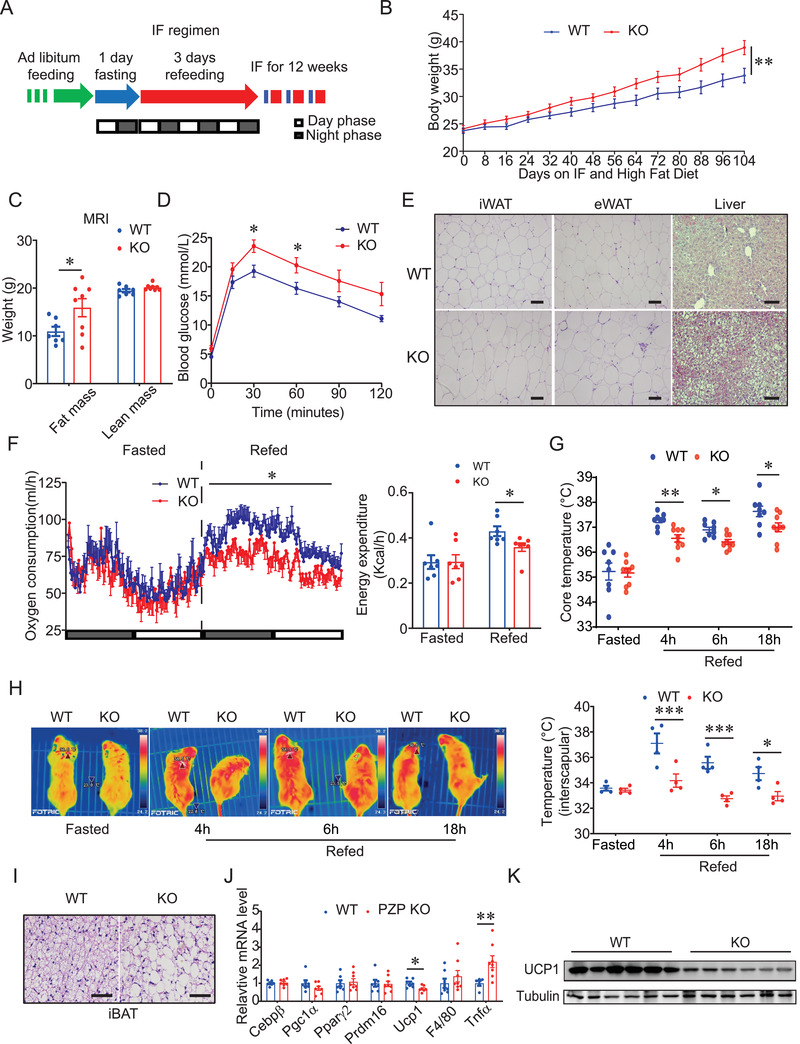
PZP plays a key role in IF‐induced metabolic remodeling. A) Schematic illustration of the 1:3 IF regimen, comprising 1 day of fasting followed by 3 days of feeding. Blue color indicating fasting; red color indicating refeeding. B) Body weight of WT and PZP KO mice is shown (*n* = 11–13). C) Body composition and D) GTT of WT and PZP KO mice when mice were fed on HFD and IF for 12 weeks is shown (*n* = 7–8). E) Representative H&E staining images of iWAT, eWAT, and liver from WT and PZP KO mice which showed PZP KO mice under IF condition had larger size of adipocytes and more lipid accumulation in the liver (scale bar = 100 µm). F) Oxygen consumption rate (left panel) and energy expenditure (right panel) of WT and PZP KO mice which showed PZP deficiency caused a marked decrease in whole‐body energy expenditure (*n* = 7). These experiments were performed when mice were fed upon HFD and IF after 2 weeks without difference in body weight between groups. G) Rectal temperature, H) surface temperature and calculated interscapular temperature of WT and PZP KO mice after fasting or indicated refeeding time (*n* = 8–9). I) Representative H&E staining images of iBAT from WT and PZP KO mice which showed PZP KO mice under IF condition had more lipid accumulation in BAT (scale bar = 100 µm). J) Relative mRNA expression of adipogenic, thermogenic and inflammatory genes in BAT from WT and PZP KO mice (*n* = 7–8). K) Immunoblots of total BAT lysates from WT and PZP KO mice which showed UCP1 level decreased in PZP KO mice using indicated antibodies (*n* = 5). Data are shown as mean ± SEM. Two‐tailed Student's *t*‐test (C,J) or one‐way ANOVA (F) or two‐way repeated measures ANOVA (B,D,H) with multiple comparisons and Tukey's post‐test were performed; *** *p* <0.001, ** *p* < 0.01, and * *p* < 0.05 were considered to be significant.

To investigate whether PZP exerts the critical roles in IF, two groups of mice were subjected to IF regimen and HFD (**Figure** **2**A). This regimen provided mice with sufficient time to compensate for the reduced amount of food intake after 1‐day fasting. No difference in cumulative food intake between IF group and non‐fasted group enabled us to examine the effects of IF, independent of caloric intake difference (Figure S3A, Supporting Information). Unlike ad libitum feeding, PZP KO mice showed a significant higher weight gain compared with WT mice when subjected to the IF regimen, thus PZP deficiency partially abolished the anti‐obesity effect of IF (Figure 2B and Figure S3B, Supporting Information). Increased adiposity in PZP KO mice was observed compared with WT mice upon IF regimen and HFD (Figure 2C and Figure S3C, Supporting Information). In addition, serum free fatty acid, cholesterol and triglyceride were significantly higher in PZP KO mice than WT mice (Table S2, Supporting Information). Glucose tolerance test (GTT) revealed that PZP deficiency in mice decreased their ability to clear exogenous glucose under IF condition (Figure 2D and Figure S3D, Supporting Information). Compared to WT mice, PZP KO mice under IF condition had larger size of adipocytes and more lipid accumulation in the liver (Figure 2E and Figure S3E–G, Supporting Information). Notably, PZP deficiency caused a marked decrease in whole‐body energy expenditure and core temperature during refeeding, compared with control mice (Figure 2F,G and Figure S3H–J, Supporting Information). However, no difference in physical activity and cumulative food intake of short‐term and long‐term were observed in PZP KO mice and WT mice (Figure S3K–M, Supporting Information).

Considering that BAT is a critical thermogenic organ and activation of BAT contributes to increased energy expenditure and anti‐obesity effects,^[^ ^3^ ^]^ we next examined the response of BAT in PZP KO mice under IF and HFD condition. We found that expression of UCP1, the key factor mediating non‐shivering thermogenesis (NST) in BAT depots, was significantly induced in BAT of refeeding mice (Figure S3N, Supporting Information), together with the elevation of PZP in liver during refeeding (Figure 1G). These data suggested that PZP may contribute to the benefits of IF through UCP1‐mediated thermogenesis. Surface temperature in the area of inter‐scapular BAT is much lower in PZP KO mice than control mice during refeeding, which revealed that PZP deficiency impaired refeeding‐induced thermogenesis of BAT (Figure 2H). Histological analysis revealed the marked enlarged lipid droplets in BAT from PZP KO mice compared with those from WT mice (Figure 2I). More importantly, PZP deficiency led the UCP1 to decrease at both mRNA and protein levels (Figure 2J,K and Figure S3Q, Supporting Information). However, we did not observe any beige adipose formation and expression changes in thermogenic genes (Pgc1*α*, Prdm16, and UCP1) in inguinal white adipose tissue (iWAT) from WT and PZP KO mice (Figure 2E and Figure S3O, Supporting Information). Expression levels of inflammation related genes (F4/80, Mcp1, and Tnf*α*) were significantly increased in adipose tissues of PZP KO mice (Figure 2J and Figure S3O,P, Supporting Information). In contrast, mRNA expression levels of genes involved in adipogenesis (Ppar*γ*2), lipolysis (Atgl and Hsl) and fatty acid oxidation (Ppar*α*) were comparable between the two groups.

Taken together, these results indicated that PZP deficiency suppressed the elevation of energy expenditure and metabolic remodeling caused by the IF dietary regimen through impairing BAT activity.

### 2.3 PZP Cell‐Autonomously Increases UCP1 Expression in Brown Adipocytes

BAT generates heat by uncoupling mitochondrial ATP synthesis, and this heat accounts for up to 20% of total energy expenditure,^[^ ^31^ ^]^ which is primarily achieved by UCP1.^[^ ^32^ ^]^

To investigate whether PZP regulates UCP1 abundance in a cell‐autonomous manner, fully differentiated primary brown adipocytes (BA) were fasted for 4 h (serum‐free DMEM contained 1 g L^−1^ glucose), then changed to refeeding medium for another 4 h supplemented with PZP protein (Figure S4A, Supporting Information). Since fetal bovine serum (FBS) contained considerable amount of bovine PZP protein that disturb our experiments, we used high glucose (9 g L^−1^) and insulin (1 um) contained DMEM as the refeeding‐mimic medium instead of FBS.

**Figure 3:**
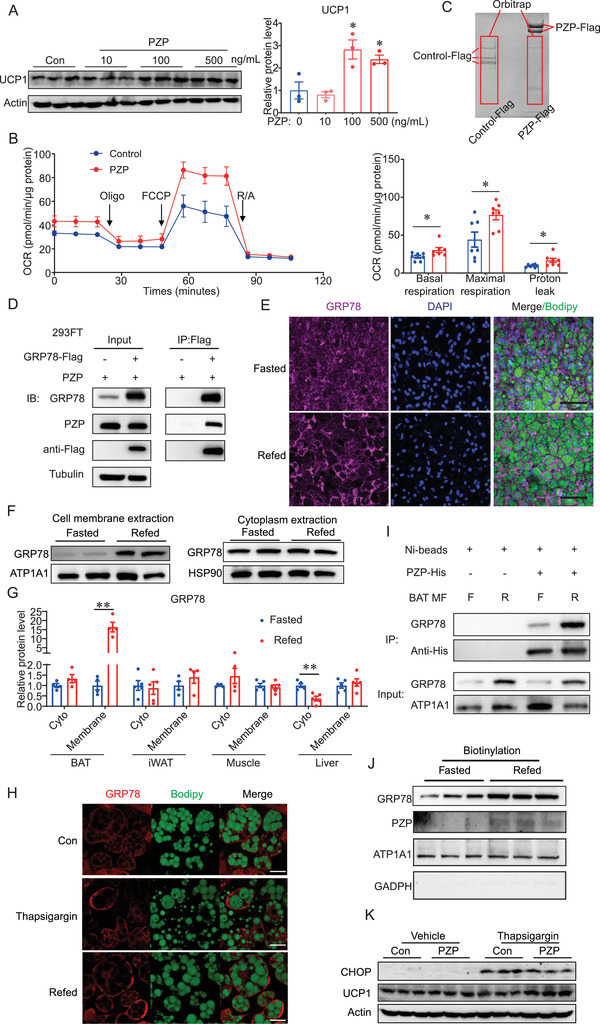
PZP promotes UCP1 expression through binding to cell surface GRP78 during refeeding. A) Immunoblots of cell lysate from differentiated BA which were cultured in refeeding medium supplemented with PZP protein (10, 100, 500 ng mL^−1^). B) Oxygen consumption of differentiated BA which were cultured in refeeding medium supplemented with PZP protein (100 ng mL^−1^) showed higher basal cellular respiration, maximal respiration, and proton leak than control group. C) SDS‐PAGE gel of anti‐Flag immunoprecipitation from 293FT cell lysate which co‐overexpressed BAT cDNA library and PZP‐Flag or Control‐Flag. Red square indicates the chopped gel which we sent for orbitrap analysis. D) 293FT was overexpressed with GRP78‐Flag and PZP. PZP‐GRP78 complex was immunoprecipitated by anti‐Flag antibody and blotted with indicated antibodies. E) Whole mount tissue staining images of iBAT from fasted and refed mice using anti‐GRP78 which showed GRP78 in BAT translocated from cytoplasm to cell membrane under IF. F) Immunoblots of cell membrane and cytoplasmic fractions from BAT using indicated antibodies which showed GRP78 dramatically increased in cell membrane fraction rather than in cytoplasm upon refeeding. G) Quantification of GRP78 protein level in cell membrane and cytoplasmic fractions of BAT, iWAT, muscle and liver from fasted and refed mice. H) Representative fluorescence images of BA which expressed GRP78‐cherry fusion protein after refeeding and thapsigargin treatment which showed refeeding and thapsigargin treatment induced translocation of GRP78 to cell surface of BA. I) Cell membrane extraction from BAT incubated with PZP‐His protein. PZP‐GRP78 complex was immunoprecipitated by anti‐His beads and blotted with indicated antibodies which showed refeeding increased GRP78‐PZP complex formation in the plasma membrane fraction of BAT. F, fasting; R, refeeding. Right panel shows result of quantitative analysis. J) Immunoblots of cell surface protein from differentiated BA which were cultured in fasting or refeeding medium supplemented with PZP protein (100 ng mL^−1^). Cell surface proteins were biotinylated and isolated by EZLabel Protein Biotin Labeling Kit and Streptavidin beads. K) Immunoblots of cell lysate from differentiated BA which were cultured supplemented with thapsigargin (600 µm) and PZP protein (100 ng mL^−1^) using indicated antibodies. Data are shown as mean ± SEM. One‐way (A) or two‐way ANOVA (B,G) with multiple comparisons and Tukey's post‐test were performed; *** *p* <0.001, ** *p* < 0.01, and * *p* < 0.05 were considered to be significant.

Interestingly, PZP protein treatment significantly increased the expression level of UCP1, which peaked at the concentration of 100 ng mL^−1^ treatment (**Figure** **3**A). Moreover, high glucose or insulin alone failed to induce the PZP‐dependent elevation of UCP1 (Figure S4B, Supporting Information). Therefore, UCP1 increased upon PZP protein treatment required a combination of glucose and insulin (Figure 3A and Figure S4B, Supporting Information). Further analysis revealed that 100 nm insulin is adequate for PZP induced UCP1 expression without affecting the differentiation efficiency (Figure S4C,D, Supporting Information). Notably, we observed that PZP increased UCP1 protein abundance gradually in the time course of refeeding and had no effect on BA, which was cultured in fasting medium (Figure S4E,F, Supporting Information). Importantly, PZP‐treated BA showed higher basal respiration, maximal respiration, and proton leak than control group (Figure 3B).

Together, these data illustrated that PZP promoted UCP1 expression in mature BA during refeeding in a cell‐autonomous manner.

### 2.4 PZP Promotes UCP1 Expression through Binding to Cell Surface GRP78 During Refeeding

The above results implied that liver‐derived PZP could activate BAT through the endocrine system. However, the detailed molecular mechanisms are still unclear. To identify the specific receptor of PZP in BAT, BAT cDNA library transfection was utilized in order to ensure the majority of receptors from BAT can express in 293FT cells (Figure S4G, Supporting Information). Then we performed a receptor capture experiment using immunoprecipitation (IP) and followed mass spectrometry. As a result, glucose‐regulated protein of 78 kDa (GRP78) emerged as a potential binding target of PZP (Figure 3C). Co‐immunoprecipitation analysis demonstrated that PZP could be co‐precipitated by GRP78 in return which confirmed PZP‐GRP78 interaction (Figure 3D).

GRP78 is a prominent endoplasmic reticulum (ER) molecular chaperone and belongs to the heat shock protein (HSP) 70 protein family. It is a multifunctional protein, and the elevation of its expression has been reported to correct the folding and assembly when cells need to initiate the unfolded protein response (UPR).^[^ ^33^, ^34^ ^]^ Although it has been recognized as an ER luminal protein, GRP78 is also found on the cell surface where it functions as a receptor for a wide variety of ligands^[^ ^35^, ^36^, ^37^ ^]^ Re‐localization of GRP78 on the cell membrane can be promoted by thapsigargin (TG), a well‐known ER stress inducer.^[^ ^38^ ^]^ To determine whether refeeding can induce the re‐localization of GRP78, we performed the whole mount tissue staining of BAT from fasted and refed mice, and as expected, we observed the translocation of GRP78 from cytoplasm to cell membrane (Figure 3E). These observations were further confirmed by the cytoplasm‐membrane fractionation experiment, which showed dramatically increased GRP78 on cell membrane rather than in cytoplasm upon refeeding and this phenomenon was BAT‐specific (Figure 3F,G and Figure S4H, Supporting Information). Consistently, similar to thapsigargin, refeeding induced translocation of GRP78 to cell surface in differentiated BA (Figure 3H). To investigate whether more GRP78 binds to PZP on the cell surface of BAT at refed condition, we performed an immunoprecipitation experiment using BAT lysates and recombinant protein. Indeed, our data showed that refeeding induced more GRP78‐PZP complex formation in the plasma membrane fraction of BAT compared to those from fasting state (Figure 3I). After the biotinylation of cell surface of BA pretreated with PZP protein, western blot analysis revealed that the refeeding induced co‐existence of GRP78 and PZP on the cell surface (Figure 3J). As described above, thapsigargin induced re‐localization of GRP78 to cell surface realized effects of refeeding (Figure 3H). Thus, the induction of UCP1 by PZP upon thapsigargin treatment without altering level of the CHOP, an ER stress marker, implied that PZP could induce UCP1 expression depending on cell membrane translocation of GRP78 during refeeding (Figure 3K and Figure S4I, Supporting Information).

### 2.5 PZP Promotes UCP1 Expression via a p38 MAPK‐ATF2 Pathway

It has been reported that cell surface GRP78 could transduce several intracellular signaling pathways, such as phosphoinositide 3‐kinase (PI3K),^[^ ^39^ ^]^ mechanistic target of rapamycin (mTOR),^[^ ^40^ ^]^ extracellular‐signal‐regulated kinase 1/2 (ERK1/2) ^41^, AMP‐activated protein kinase (AMPK),^[^ ^42^ ^]^ and p38 MAPK.^[^ ^41^ ^]^ It is of interest to explore the GRP78 downstream pathway in BAT that induces the expression of UCP1 at the condition of refeeding.

**Figure 4:**
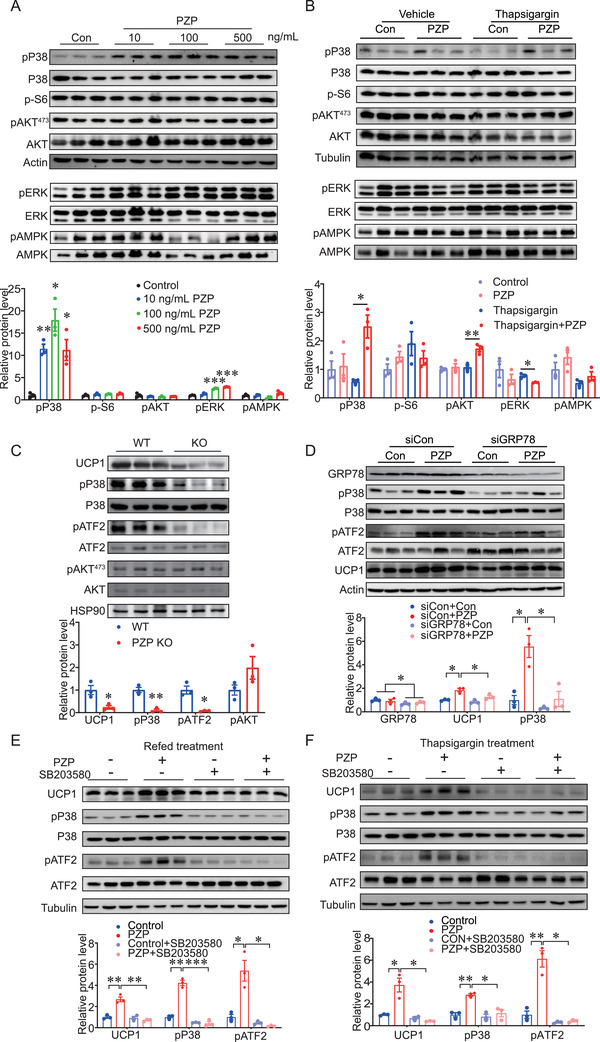
PZP promotes UCP1 expression via a P38 MAPK‐ATF2 pathway. A) Immunoblots of cell lysate from differentiated BA which were cultured in refeeding medium supplemented with PZP protein (10, 100, 500 ng mL^−1^) using indicated antibodies which showed PZP significantly increased the phosphorylation of p38 MAPK in condition of refeeding. B) Immunoblots of cell lysate from differentiated BA which were cultured supplemented with or without thapsgargin (600 µm) and PZP protein (100 ng mL^−1^) using indicated antibodies. C) Immunoblots of BAT lysates from control mice and PZP KO mice when mice were fed upon HFD and IF after 2 weeks without difference in body weight between groups using indicated antibodies which showed phosphorylation of p38 MAPK and ATF2 were decreased in PZP KO mice. The samples were collected when mice were refed for 6 h after 24‐h fast. D) Immunoblots of cell lysate from siGRP78‐transfected BA and controls which were cultured in refeeding medium supplemented with or without PZP protein (100 ng mL^−1^) using indicated antibodies, which showed UCP1 expression and phosphorylation of p38 MAPK and ATF2 upon PZP treatment were significantly inhibited by GRP78 siRNA. E) Immunoblots of cell lysate from differentiated BA which were cultured in refeeding medium supplemented with or without SB203580 (10 µm) and PZP protein (100 ng mL^−1^) using indicated antibodies, which showed PZP‐induced UCP1 expression under refeeding condition was significantly inhibited by SB203580 treatment. F) Immunoblots of cell lysate from differentiated BA which were cultured supplemented with or without SB203580 (10 µm) and PZP protein (100 ng mL^−1^) after thapsigargin (600 µm) stimulation using indicated antibodies, which showed PZP induced UCP1 expression under thapsigargin treatment was significantly inhibited by SB203580 treatment. Data are shown as mean ± SEM. One‐way (A–C) or two‐way ANOVA (D–F) with multiple comparisons and Tukey's post‐test were performed; *** *p* <0.001, ** *p* < 0.01, and * *p* < 0.05 were considered to be significant.

Thus, we examined the well‐known GRP78‐mediated pathways, including mTOR, PI3K, ERK1/2, and AMPK in differentiated BA upon PZP treatment by Western blot. Our data showed that PZP significantly increased the phosphorylation of p38 MAPK in both refeeding (**Figure** **4**A) and thapsigargin treatment (Figure 4B), while the phosphorylation of ribosomal protein S6 (mTOR signaling), AKT473 (PI3K signaling) and ERK1/2 and AMPK was not affected (Figure 4A,B). It has been reported that the activated p38 MAPK‐ATF2 axis increases UCP1 expression.^[^ ^43^, ^44^ ^]^ In line with these observations, phosphorylation of p38 and ATF2 was impaired in PZP KO mice (Figure 4C). Furthermore, UCP1 expression and phosphorylation of p38 and ATF2 upon PZP treatment were significantly inhibited by GRP78 siRNA (Figure 4D). In addition, SB203580, a p38 MAPK inhibitor, was supplemented into the medium during PZP treatment, then we observed that the PZP induced UCP1 expression was significantly inhibited in either refeeding or thapsigargin treatment (Figure 4E,F). Collectively, these data indicated that p38 MAPK‐ATF2 signaling is crucial for induction of UCP1 via PZP‐GRP78 interaction during refeeding condition.

### 2.6 PZP Recombinant Protein Protects Mice from DIO During IF Treatment

**Figure 5:**
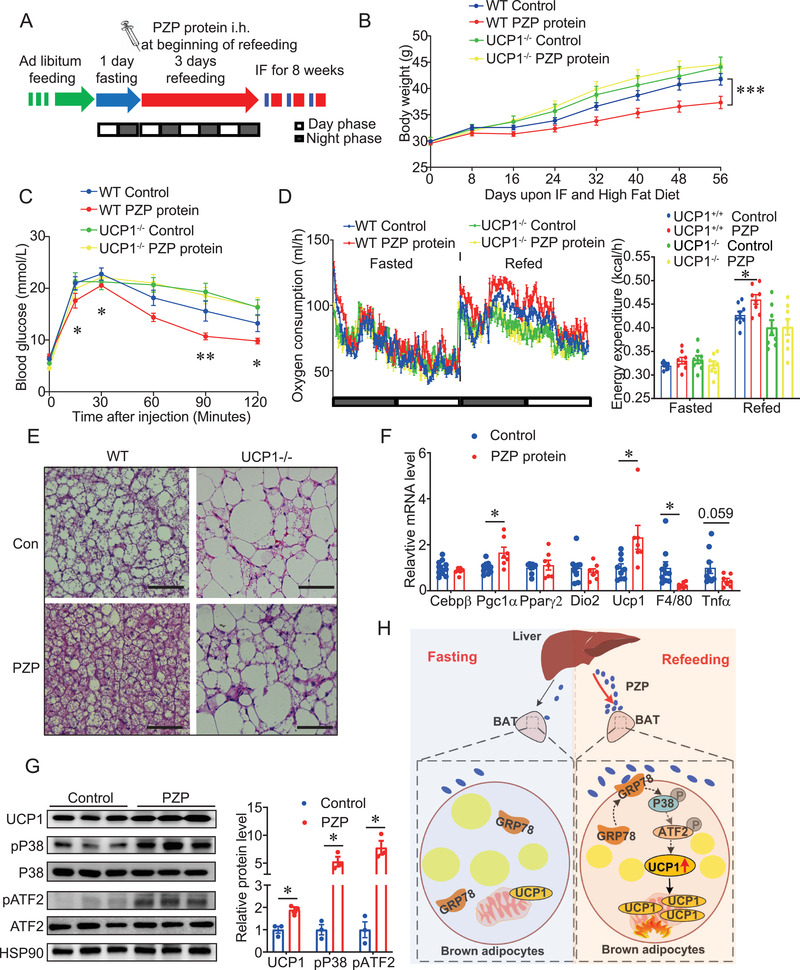
Increased circulating PZP augments UCP1‐dependent energy expenditure and protects DIO during IF treatment WT and UCP1−/− mice chronically subjected to HFD for 7 weeks. A) Schematic shows the procedure of in vivo therapeutic experiments. Mice were fed in IF regimen, comprising 1 day of fasting followed by 3 days of feeding per cycle for 8 weeks. We performed i.h. of PZP protein at the beginning of refeeding. Blue color indicating fasting; red color indicating refeeding. B) Body weight and C) GTT of WT and UCP1−/− mice treated with or without PZP protein (1 mg/kg) are shown (*n* = 8–10). D) Oxygen consumption rate (left panel) and energy expenditure (right panel) of WT and UCP1−/− mice treated with or without PZP protein (*n* = 8–9). These experiments were performed when mice were upon HFD and IF after 2 weeks without difference in body weight between groups, which showed PZP protein injection dramatically increased the oxygen consumption and energy expenditure of WT mice but not UCP1^−/−^ mice. E) Representative H&E staining images of iBAT from WT and UCP1−/− mice treated with or without PZP protein (scale bar = 100 µm). F) Relative mRNA expression of adipogenic, thermogenic, and inflammatory genes in BAT from WT mice treated with or without PZP protein (*n* = 7–10). G) Immunoblots of total BAT lysates from WT mice treated with or without PZP protein using indicated antibodies which showed PZP protein treatment activated p38 MAPK‐ATF2‐UCP1 signaling in WT mice (*n* = 3). H) Schematic overview of main findings. Refeeding signals induced liver to produce and release PZP into circulation and followed by the translocation of GRP78 to cell surface in BAT. Subsequently, circulating PZP could bind to cell surface GRP78 to promote UCP1 expression via a p38 MAPK‐ATF2 signaling pathway in BAT. Data are shown as mean ± SEM. Two‐tailed Student's *t*‐test (F,G) or two‐way repeated measures ANOVA (B–D) with multiple comparisons and Tukey's post‐test were performed; ***p<0.001, ** *p* < 0.01, and * *p* < 0.05 were considered to be significant.

To explore the therapeutic potential of PZP for DIO, PZP recombinant protein was hypodermic injected at the beginning of refeeding time in WT and UCP1^−/−^ mice (**Figure** **5**A). The injection dose was optimized first, and we found that a high dose (1 mg/kg body weight) of PZP protein efficiently increased the expression level of UCP1 and activated p38 MAPK‐ATF2 signaling in BAT of WT mice during refeeding but not fasting state (Figure S5A–D, Supporting Information). More importantly, we examined the in vivo pharmacokinetics profile of PZP protein using a self‐made sandwiched ELISA kit. We observed that serum PZP level reached a peak 2 h after a single protein injection and maintained at a high concentration for at least two days (Figure S5E, Supporting Information). Our data showed that PZP protein administration significantly reduced the body weight gain compared to those from control group in the long‐term experiment. However, this beneficial effect of PZP was totally abolished in UCP1 KO mice (Figure 5B). Moreover, reduction of adiposity and fat accumulation in liver was observed in WT mice but not in UCP1^−/−^ mice after PZP protein injection (Figure S5F,K, Supporting Information). Serum‐free fatty acid, cholesterol, and triglyceride were significantly lower in WT mice but not UCP1−/− mice after PZP protein injection (Table S2, Supporting Information). In addition, PZP protein injection significantly improved the glucose tolerance (Figure 5C and Figure S5G, Supporting Information) and dramatically increased the oxygen consumption and energy expenditure (Figure 5D and Figure S5H–J, Supporting Information) of WT mice but not UCP1^−/−^ mice during the refeeding state, without changes in physical activity and food intake (Figure S5L,M, Supporting Information). More importantly, PZP protein administration attenuated the hypertrophy of BAT (Figure 5E) and increased UCP1 level in WT mice (Figure 5F,G). As expected, p38 MAPK signaling was also activated upon PZP protein treatment in WT mice (Figure 5G). To confirm the therapeutic effect of PZP in established‐obese mice, PZP KO mice were subjected to IF regimen and kept on HFD after 12‐week HFD. We found that PZP protein administration significantly reduced the body weights of PZP KO mice, from 45.33 ± 1.47 g to 40.22 ± 1.15 g, corresponding to a 10.95% ± 1.95% weight loss relative to their initial weights (Figure S6A,B, Supporting Information). The body weights of vehicle‐treated PZP KO mice showed no changes, and PZP protein did not affect cumulative food intake (Figure S6E, Supporting Information). In addition, PZP protein injection significantly improved the glucose tolerance in PZP KO mice (Figure S6C, Supporting Information). Moreover, reduction of adiposity, lipid accumulation in the liver and serum lipids were observed in PZP KO mice after PZP protein injection (Figure S6D,H and Table S2, Supporting Information). As expected, PZP treatment increased UCP1 protein level and significantly activated p38 MAPK‐ATF2 signaling in PZP KO mice (Figure S6F,G, Supporting Information). The above results imply that refeeding‐induced PZP may play a critical role in IF‐induced metabolic remodeling in a UCP1‐dependent manner.

### 2.7 Anti‐Obesity Effect of IF is Impaired in PZP^Δliver^ Mice but Rescued by PZP Protein

To confirm the essential role of liver‐derived PZP in the anti‐obesity effect, we generated a liver‐specific PZP deficiency model (PZP^Δliver^) based on adeno‐associated viral (AAV) CRISPR/Cas9 strategy,^[^ ^45^ ^]^ where sacas9 expression is driven by liver‐specific thyroxine‐binding globulin (TBG) promoter (Figure S7A, Supporting Information). Injection of AAV efficiently caused a 55% decrease of PZP expression in liver from PZP^Δliver^ mice but not in other tissues (Figure S7B, Supporting Information). PZP protein was almost deleted in liver and serum from PZP^Δliver^ mice due to cas9‐driving gene editing (Figure S7C, Supporting Information). These results are similar to the previous report, which showed transcription level of Hao1 was reduced, and the protein level was remarkably deleted using the same strategy.^[^ ^46^ ^]^

The strategy of IF regimen and HFD was also used in PZP^Δliver^ mice. PZP^Δliver^ mice showed a significant higher weight gain compared with PZP^scramble^ mice (Figure S7D, Supporting Information). Increased adiposity in PZP^Δliver^ mice was observed when compared with PZP^scramble^ mice (Figure S7E, Supporting Information). GTT revealed that liver‐specific PZP deficiency in mice decreased their ability to clear exogenous glucose under IF condition (Figure S7F, Supporting Information). Notably, PZP deficiency in liver caused a marked decrease in whole‐body energy expenditure during refeeding, compared with PZP^scramble^ mice (Figure S7G–J, Supporting Information). Compared to PZP^scramble^ mice, PZP^Δliver^ mice under IF condition had a larger adipocyte size and more lipid accumulation in the liver (Figure S7K, Supporting Information). Notably, PZP deficiency in liver caused a marked decrease in UCP1 protein level (Figure S7L, Supporting Information). However, all the demerits of PZP deficiency were rescued by exogenous PZP protein injection (Figure S7D–L, Supporting Information). According to these data, hepatic PZP was indeed the main contributor to circulating PZP, which played a critical role in IF‐induced metabolic remodeling.

## 3 Discussion

Inter‐organ crosstalk of whole‐body energy balance,^[^ ^47^ ^]^ especially of the BAT activation mediated anti‐obesity,^[^ ^48^ ^]^ is a new frontier in the field. However, whether the liver promotes BAT thermogenesis via hepatokines remains poorly understood. In the present study, we define a previously unrecognized cross‐organ communication system that IF improves whole‐body metabolism through liver‐BAT interaction, which is critical for IF induced metabolic remodeling. Further, we found that fasting‐refeeding signals induced the liver to produce and release PZP into circulation and followed by the translocation of GRP78 to the cell surface in BAT. Then PZP‐GRP78 interaction promotes UCP1 expression via p38 MAPK‐ATF2 signaling (Figure 5H).

In the current study, we revealed PZP as a key hepatokine regulating IF triggered energy homeostasis through liver/BAT axis. In support of our notion, another hepatokine, Tsukushi (TSK), was also involved in metabolic regulation through liver/BAT axis. Interestingly, TSK, which is highly inducible in response to NAFLD^[^ ^49^ ^]^ or high energy expenditure,^[^ ^50^ ^]^ inhibits the BAT activity acting as a suppressor of thermogenesis.^[^ ^50^ ^]^

This is the first study demonstrating that p38 MAPK‐ATF2 signaling is crucial for induction of UCP1 via PZP‐GRP78 interaction during metabolic remodeling. Consistent with these observations, PZP deficiency partially lost anti‐obesity effects during IF, which relied on UCP1‐dependent thermogenesis. Furthermore, PZP protein injection could augment this IF‐induced metabolic remodeling, raising the prospect of developing PZP as an effective therapeutic target for obesity and diabetes. Since diverse outcomes of weight loss usually occurred in clinical studies, it is conceivable that the serum level of PZP in individual might predicts the anti‐obesity outcome of IF.

PZP was considered as pregnancy zone protein because of its dramatically elevated expression during human gestation.^[^ ^51^ ^]^ However, the studies about the physiological role of PZP associated with pregnancy are controversial and remain unclear: i) Similar to alpha‐2‐macroglobulin (A2M), PZP was regarded as a protease inhibitor, but PZP only inhibits a narrow spectrum of proteases and has no correlation with pregnancy;^[^ ^16^ ^]^ ii) As an inhibitor of the immune response, PZP is capable of protecting fetus from maternal immune system attack;^[^ ^52^ ^]^ iii) Recently, Cater et, al. has reported that PZP is able to inhibit the aggregation of misfolded proteins, which usually accumulate in late pregnancy and many age‐related disorders such as Alzheimer's disease (AD), arthritis, and atherosclerosis.^[^ ^53^ ^]^ Given the fact that the successful gestation requires remarkable increase in energy intake,^[^ ^54^ ^]^ it is conceivable that PZP plays a critical role in maintaining the maternal metabolic homeostasis during pregnancy. Thus, we propose another feasible explanation that how PZP works.

A series of preclinical studies have proved that IF could efficiently delay the onset and progression of AD processes in animal models.^[^ ^55^ ^]^ Combined with Cater's study and our data, IF‐induced PZP inhibits the aggregation of misfolded proteins in the brain, which might partially explain this inherent mechanism. However, more extensive future studies are needed to explore this possibility.

Recently, PZP has been described as a novel biomarker for screening lung adenocarcinoma in T2DM patients or predicting the prognosis of hepatocellular carcinoma.^[^ ^56^, ^57^ ^]^ However, It was noted that PZP expression is lower in tumor tissues than that in normal tissues^[^ ^58^, ^59^ ^]^ and ablation of PZP promotes breast cancer progression.^[^ ^60^ ^]^ Thus, our protein therapy is safe without adverse effect in promoting tumor growth.

This study utilized a novel screening method to identify potential hepatokines that function in energy homeostasis upon over‐nutrition. Among proteins that we screened, a few proteins (e.g., Afm, Masp1, and Serpina12) were found to be closely related to metabolism homeostasis, which indirectly supports our results that PZP is a metabolic response protein. Serpina12, also called vaspin, was able to reduce HFD‐induced weight gain and improve glucose tolerance and hepatic steatosis.^[^ ^42^ ^]^ Interestingly, the receptor of vaspin is also GRP78. Accumulating reports provide evidence for a novel homeostatic function of GRP78 in mediating fatty acid oxidation and steroidogenesis.^[^ ^61^, ^62^ ^]^ Specific overexpression of GRP78 in hypothalamus could revert HFD‐induced obesity by promoting BAT thermogenesis and beige formation in iWAT.^[^ ^63^, ^64^ ^]^ However, our study demonstrated that cell membrane translocation of GRP78 played a role in releasing the metabolic stress of over‐nutrition. Previous reports have demonstrated that ER stress could promoted the re‐localization of GRP78 on the cell membrane^[^ ^65^, ^66^, ^67^ ^]^ but our data showed a combination of glucose and insulin is another potential inducer for translocation of GPR78 to cell surface. This phenomenon is very interesting, but the in‐depth mechanism needs to be further explored.

In summary, we herein identified a new inter‐organ crosstalk system between liver and BAT during metabolic remodeling. In addition, we revealed that the circulating PZP increased UCP1 expression in BAT through binding to cell surface GRP78 via the p38 MAPK‐ATF2 signaling pathway. Future clinical studies are needed to explore the therapeutic potential of PZP for the development of precision medicine to treat obesity and related diseases.

## 4 Experimental Section

### Mice

All animal studies were approved by the Institutional Animal Care and Use Committee of the Institute of Zoology, Chinese Academy of Sciences. 4–6 weeks old C57BL/6 mice were purchased from Vital River Laboratory Animal Technology Co Ltd. PZP‐knockout mice were generated using the CRISPR/Cas9 system. Guide RNAs flanking the exon 1 and exon 3 of PZP (usgRNA1: GGGCAGCTGGTTTCTCCTCA, dsgRNA2: GTAATGAATGTGTAGGGTAA) were microinjected with Cas9 mRNA into fertilized eggs of C57BL/6 background mice at the Institute of Zoology, Chinese Academy of Sciences. UCP1 KO mice were obtained as a gift from L Kozak (Pennington Medical Research Centre, Baton Rouge, Louisiana, USA).^[^ ^68^ ^]^ Heterozygous PZP knockout mice intercrossing was adopted to generate PZP KO and littermate WT control mice. Heterozygous UCP1 knockout mice were intercrossed to obtain UCP1 KO mice and littermate wide type control mice. All the mice were housed in our SPF laboratory animal‐house (Institute of Zoology of Beijing, Chinese Academy of Sciences, China) at 24 °C room temperature with a 12‐h light/dark cycle; five mice were housed in each cage with free access to water for all experiments. The normal chow diet is composed of 11.7% kcal from fat, 66.1% kcal from carbohydrates and 22.2% kcal from protein (MD17121, medicience). HFD is composed of 60% from fat, 20% kcal from carbohydrates and 20% from protein (MD12033, medicience).

WT and PZP KO mice were fed on chow diet for 15 weeks and fed on high fat diet for 11 weeks. WT and PZP KO mice were fed on HFD and IF for 104 days. WT and UCP1^−/−^ mice were fed on HFD and IF for 56 days. PZP^scramble^ and PZPΔliver mice were fed on HFD and IF for 56 days. PZP KO DIO mice were fed on HFD and IF for 72 days.

### Human Samples for Detection of PZP Abundance

Human protocol was approved by Institutional Review Board of Huashan Hospital at Fudan University (HIRB, The certificate no. 2016395). The BMI associated blood samples were donated by participants who were randomly selected from 20–50 years male (*n* = 15) or female (*n* = 29) volunteers. Blood samples were collected and then isolated after centrifuged at 3000 rpm at four degree for 10 min. The fasted and refed human blood samples were provided by volunteers participating in formal OGTT. In brief, after 12–14 h of fasting, the volunteers were given 75 g glucose orally. With the consent of the volunteers, blood samples were collected before and 2 h after the glucose administration. Human protocol was approved by Institutional Review Board of Huashan Hospital at Fudan University (HIRB, The certificate no. 2016395).

### Searching for Hepatic Secreted Factors Related to Metabolism

To find the hepatokines which are related to metabolism, the following publicly available datasets were used for the screening: liver‐specific enriched expression compared with any other tissues (NCBI GEO: GSE9954), increased expression during feeding compared with that during fasting in the liver (NCBI GEO: GSE46495) and decreased expression on HFD compared with that on chow diet in the liver (NCBI GEO: GSE35961). Thirty‐five potential proteins were tagged in the overlap of the above three criteria.

### Glucose Tolerance Tests

After the last IF cycle, mice were subjected to an intraperitoneal glucose tolerance tests (IPGTT). IPGTT was performed as follows: after food deprivation for 16 h, mice were intraperitoneally injected with glucose solution. To induce an adequate insulin response, lean mice on chow diet were injected intraperitoneally with glucose (2.0 g/kg body weight),^[^ ^69^, ^70^ ^]^ and glucose (1.5 g/kg body weight) was administrated for DIO mice^[^ ^71^, ^72^ ^]^ due to the glucose intolerance of obese mice and the detection limits of the glucose meter. Plasma glucose was measured with a glucose meter (Roche Diagnostics Corp) before and 15, 30, 60, 90, and 120 min after injection.

### Histological Analysis

For H&E staining, tissues fixed with 4% Paraformaldehyde were embedded in paraffin. 5‐µm‐thick sections were stained and observed under 10× or 20× objective lens. For whole mount staining, BAT was dissected from mice inter‐scapular and incubated on ice with 2% PFA overnight, following by permeable treatment with 0.3% Triton ×‐100 and 3% FBS in PBS for 1 h in RT. After that, tissues were removed into staining buffer containing primary antibody (anti‐GRP78, ab21685) in 4 °C for 24 h, followed by incubation with a fluorophore‐conjugated secondary antibody. Then the whole BAT was scanned under a confocal laser‐scanning microscope (Zeiss LSM 780).

### Real‐Time qPCR

Total RNA from tissues and cells was isolated with TRIzol Reagent (Thermo Fisher Scientific, USA) and reverse‐transcribed with high‐capacity cDNA reverse transcription kit (Promega, USA). The relative expression of genes was detected by real‐time fluorescence quantitative polymerase chain reaction (Light Cycler 480, Roche, Sweden) with SYBR Green Master Mix (Promega, USA). The list of primers are shown in Table S1, Supporting Information.

### Western Blot

For relative protein expression level detection, a series of western blot were performed. Briefly, total proteins of cells were extracted by RIPA buffer and tissue proteins were obtained by additional chopping with a T10 basic ULTRA‐TURRAX handheld homogenizer (IKA, Germany). Equal amount of proteins were loaded in 10% SDS–polyacrylamide gels to get separated and then were transferred to PVDF membranes. The membranes with proteins were incubated with the following antibodies overnight in 4 °C: anti‐PZP (LS‐C296025, Lifespan), anti‐UCP1 (ab155117, Abcam), anti‐GRP78 (ab21685, Abcam), anti‐phospho ATF2 (ab32019, Abcam), anti‐PPAR*α* (2443S, CST), anti‐CHOP (2895S, CST), anti‐ERK (9102S, CST), anti‐phospho ERK (9101S, CST), anti‐AMPK (2603S, CST), anti‐phospho AMPK (2535S, CST), anti‐AKT (9272S, CST), anti‐phospho AKT473 (9271S, CST), anti‐p38 MAPK (9212S, CST), anti‐phospho p38 MAPK (4631S, CST), anti‐ATF2 (35031S, CST), anti‐ATP1A1 (55187‐1‐AP, Proteintech), anti‐Flag (F1804, Sigma), anti‐Tubulin (2146, CST), anti‐Actin (3700, CST), anti‐GAPDH (5174, CST), anti‐His (9991, CST), then were incubated with HRP‐conjugated secondary antibodies for 1 h at room temperature. All signals were visualized and analyzed by densitometric scanning (Image Quant TL7.0; GE Healthcare Biosciences, Uppsala Sweden). Intensity values of the bands were analyzed by using ImageJ software (National Institutes of Health, Bethesda, MD, USA).

### Production and Purification of PZP Protein

For PZP protein production, the mouse PZP C‐terminal subunit sequence (35 kDa receptor binding domain, 771bp^[^ ^73^ ^]^) together with a 6× His‐tag was cloned into pmal‐C5x construct. The construct was transformed into BL21 (DE3) cells and induced with 0.4 mg L^−1^ IPTG for 16 h at 24 °C. The cultured cells were harvested by centrifugation and re‐suspended in PBS buffer. To prepare the supernatant fraction, cells in PBS buffer was broken by ultrasonic and centrifuged at 12 000 rpm for 20 min at 4 °C. MBP‐PZP‐His recombinant protein in the supernatant was purified with HisTrap FF affinity chromatography, and then was eluted with PBS buffer containing 1 m imidazole and final kept in cleaning PBS buffer through dialysis. MBP tag protein expressed by pmal‐C5x vector was purified as control protein. For in vivo therapeutic experiment, 10 weeks WT and UCP1 −/− mice were fed upon HFD and IF diet regimen after one‐week high fat diet adaptation, then were hypodermically (i.h.) injected with PZP‐MBP or MBP protein at doses of 1 mg/kg at the beginning of the refeeding day.

### Pharmacokinetic Tests

Purified recombinant MBP‐PZP protein was used as a standard substance. 1 mg/kg MBP‐PZP protein was subcutaneously injected into mice, and serum was collected from the tail vein at a series of time points. 100 µL/well diluted MBP antibody (NEB, E8032) in coating buffer (0.05 m carbonate buffer, pH 9.5, 1:500) was added in 96‐well ELISA plate at 4 °C overnight. Plates were washed four times with 220 uL/well TBST, then were blocked with 200 uL/well 2% BSA for 2 h at room temperature. After washing, 100 uL/well standards and diluted serum were added in the plates. The plates were incubated at 37 °C for 1 h. After washing, 100 uL/well of the detector antibody (LS‐C296025, Lifespan, 1:1000) diluted in TBST were added to the plates and the plates were incubated for 1 h at 37 °C. After washing, the plates were coated with 100 uL/well TMB substrate reagent set (Tiangen Biotech) and incubated for 30 min at room temperature in the dark. The reaction was stopped by adding 50 uL/well 2 m H_2_SO_4_, and the absorbance was read at 450 nm.

### Metabolic Rate and Physical Activity

Oxygen consumption was measured when mice were fed upon HFD for 2 weeks without difference in body weights between groups and physical activity was determined at 12 weeks of age with a TSE LabMaster (TSE Systems, Bad Homburg, Germany), as previously described.^[^ ^74^ ^]^ Mice were acclimated to the system maintained at 24 °C in a 12‐h light/dark cycle for 20–24 h, and fasted VO_2_ and VCO_2_ of each mouse were measured during the next 24 h with food deprivation. Then refed VO_2_ and VCO_2_ were obtained with free access to food and water. The voluntary activity of each mouse was measured with an optical beam technique (Opto‐M3, Columbus Instruments, Columbus, OH, USA) over 24 h and expressed as 24‐h average activity. Oxygen consumption and energy expenditure data were analyzed using CaIR (version 1.1) (https://calrapp.org/).^[^ ^75^ ^]^

### Body Composition Measurements

The total fat and lean mass of mice were assessed with the Small Animal Body Composition Analysis and Imaging System (MesoQMR 23 060H‐I; Nuimag Corp., Shanghai, China), according to the manufacturer's instructions.

### Cold Challenge Experiment

A cold tolerance test was performed with PZP TG and control mice. Mice were placed in a cold chamber (4 °C) for up to 4 h with free access to food or water. Body temperature was measured using a rectal probe connected to digital thermometer (Yellow Spring Instruments).

### Temperature Measurements

Skin temperature surrounding BAT was recorded with an infrared camera (E60: Compact Infrared Thermal Imaging Camera; FLIR; West Malling, Kent, UK) and analyzed with a specific software package (FLIR ResearchIR Max 3.4; FLIR; West Malling).

### Cell Culture Experiments

293FT cell line was obtained from the ATCC and was cultured in Dulbecco's modified Eagle medium (DMEM, Gibco, USA) with 10% FBS (Gibco, USA) at 37 °C. For stable transfection, the 3‐plasmid lentivirus‐mediated gene transfer and expression system were used. Briefly, core plasmid of pCDH‐EF1‐MCS‐T2A‐copGFP, packaging plasmid of psPAX2 and envelope plasmid of pMD2.G were mixed at the ratio 4: 3: 2 in DMEM, with threefold volume of PEI co‐incubated for 20 min at room temperature. The mixture was added into cell‐culture dish which contains 70–90% confluent 293FT cells for 6 h, and then was replaced by fresh basal culture medium. 48 h later, a cell culture supernatant, which contained packaged lentivirus particles was collected.

A brown stromal vascular fraction was isolated from C57BL/6J neonatal mice according to standard procedures. Briefly, 6–8 newborn mice were disinfected with 75% alcohol. The interscapular brown fat pad was separated and cut into 1 mm^[^ ^3^ ^]^ particles, after that, the minced tissues were transferred into a 1.5 mL tube containing 1 mL digestion buffer (1.5 mg mL^−1^ collagenase I) and incubated in 37 °C water bath for digesting. Digestion was stopped by adding 5 mL of culture medium and the mixture was filtered through a 100 µm cell strainer into a new 50 mL sterile tube followed with centrifuging at room temperature at 600× g for 5 min. The SVF pellet was re‐suspended and cultured with DMEM medium containing 20% FBS in a 37 °C 5% CO_2_ incubator. For BA differentiation assays, 100% confluent SVF was changed into the induction medium which contained 0.5 mm 3‐isobutyl‐1‐ methylxanthine, 2 µg mL^−1^ dexamethasone, 20 nm insulin, 1 µm rosiglitazone, 1 nm 3,3′,5‐triiodo‐l‐thyronine (T3) and 0.125 mm indomethacin. Two days later, the medium was changed by maintaining medium which contained 20 nm insulin, 1 µm Rosiglitazone and 1 nm T3 and continued until day 6.

To mimic fasting‐refeeding condition, the differentiated BA were cultured in serum‐free low glucose DMEM (1 g L^−1^ glucose) for 4 h, after that, the refed medium, DMEM with 100 nm insulin, 9 g L^−1^ glucose was changed into the cell culture dish. In addition, 10 µm SB203580 or DMSO was added in refed medium to inhibit phosphorylated p38 MAPK when needed. 600 µm thapsigargin was added in serum‐free DMEM to induce translocation of GRP78 to cell surface.

To visualize the membrane translocation of GRP78 in BA during refeeding, immunofluorescence staining was performed. Briefly, BAT primary cells were seeded in confocal dishes and differentiated into mature BA. On the last differentiation day, cells were changed with serum‐free low glucose DMEM (fasted group). 4 h later, several dishes of cells were treated with mimic medium (refed group) or thapsigargin (600 µm) for another 1 h. Then cells were fixed with 4% paraformaldehyde and perforated with 0.5%Triton ×‐100. Cells were dyed with anti‐GRP78 antibody, and anti‐rabbit secondary antibody in conjunction with TRITC (red) and BODIPY dyes (green) and then were scanned with a confocal microscopy (LSM 710; Carl Zeiss Co., Ltd.)

To measure cellular respiration, BA were seeded on a gelatin‐pretreated seahorse cell culture‐plate and were differentiated for 6 days. The differentiated BA were cultured in serum‐free low glucose DMEM (1 g L^−1^ glucose) for 4 h, then changed into feeding mimic medium with additional 500 ng mL^−1^ MBP or MBP‐PZP fusion protein. O_2_ consumption of treated adipocytes was measured with an XF24‐3 extracellular flux analyzer (Agilent Technologies, Santa Clara, CA, USA).

For GRP78 knock‐down assays in primary adipocytes, the following specific small interference RNA sequences were used: siRNA‐targeting mouse GRP78 mRNA (sense strand, 5′‐ GCGUCGGUGUGUUCAAGAACG‐3′) and negative control, (5′‐UCUCCGAACGUGUCACGUTT‐3′). When primary BA were cultured to 70% confluent, 50 nm siGRP78 or an equal amount of control siRNA was added into the medium with LipofectamineTM 2000 Transfection Reagent (Invitrogen, 11668–019) according to the reagent instruction. After getting confluent, transfected cells were used for differentiation and further treatment in 10 days.

An 8‐week‐old C57BL/6 strain of mouse was anesthetized and secured via pining four limbs to isolate primary liver cells. Abdominal cavity was carefully dissected to expose liver, portal vein (PV), and inferior vena cava (IVC) without nicking any internal organs. Heart entrance was interrupted with a hemostatic forceps, and a hose needle was fixed in PV with another hemostatic forceps. 50 mL HBSS was injected into PV via the hose needle at a constant rate and the IVC was cut to washing liver, then 50 mL digestion medium (DMEM‐low glucose with 1× Penn‐Strep and 15 mm HEPES) containing 0.5 mg mL^−1^ Type IV collagenase was injected within 30 min. The liver was pulled free and placed in a 10 cm dish containing digestion medium. When the liver began to swell, the lobes of the liver was torn apart, and shook gently to free residual cells. The suspension was filtered through a 70–75‐micron membrane, adding 40 mL isolated medium (50/50 DMEM‐High/F‐12 containing 10% FBS), and then spun at 4 °C for 2 min at 50 × g in a swinging‐arm centrifuge for three times. Cells were cultured into a 10 cm dish at 300 000 cells mL^−1^ cell density according to a trypan blue counting.

### Plasma Membrane Fraction Isolation

To prepare cell membrane fractions of BAT, a plasma membrane protein isolation kit (SM‐005, invent) was used. Firstly, total membrane proteins were isolated. Briefly, tissues were physically grinded in buffer A and were high‐speed centrifuged (14,000 rpm for 30 s), the deposit were re‐suspended and centrifuged at 3000 rpm for 1 min (the pellet contains intact nuclei). The supernatant was transferred to a fresh 1.5 mL micro centrifuge tube and centrifuged at 4 °C for 30 min at 16 000 × g. Remove the supernatant (this is the cytosol fraction) and save the pellet (this is the total membrane protein fraction including organelles and plasma membranes). And then cell membrane proteins were further isolated from total membrane proteins using differential velocity centrifugation.

To confirm PZP‐GRP78 complex in plasma membranes, cell surface proteins of BA were biotinylated and isolated by Sulfo‐NHS‐Biotin reagent (Apexbio) and BiotSep Streptavidin 6FF Chromatography Column (20514ES08, Yeasen).

### Screening and Verification of PZP Receptor

The coding sequence of the mouse PZP gene was cloned into pCDH‐EF1‐MCS‐T2A‐copGFP plasmid together with a Flag‐tag. 293FT cells that stably expressed PZP‐flag were obtained by flow cytometer screening for GFP‐positive signal after 48‐h infection. To find the potential receptor of PZP in BAT, a BAT cDNA library was transiently transfected into above 293FT cells for 48 h. The cell lysate from mentioned co‐overexpression cell was incubated with anti‐Flag affinity gel (20585ES03, Yeasen) overnight. The binding protein complexes were eluted by 0.1 m Gly‐HCl (pH 3.5) after three times washing with TBS buffer (50 mm Tris‐HCl, pH 7.4, 150 mm NaCl). The eluted protein complexes from two groups were loaded in a SDS‐PAGE gel. After electrophoresis, the gels including proteins from 20 to 160 kDa were identified with liquid chromatography with linear ion trap‐orbitrap mass spectrometry (LTQ/Orbitrap‐MS) (LTQ Orbitrap XL, mass spectrometry group, Institute of Biophysics, CAS), and the potential receptor were selected according to previous literature reports and characteristic of the receptor. To further determine whether GRP78 could bind directly with PZP, the coding sequence of the mouse GRP78 gene was cloned into a pCDH‐EF1‐MCS‐T2A‐copGFP plasmid with a Flag‐tag, and then GRP78 was stably expressed in 293FT cells via the same method. An untagged PZP overexpressing plasmid was transiently transfected into 293FT cells, which GRP78‐flag stably expressed. After 48 h, the cell lysate was incubated with anti‐DYKDDDDK (Flag) affinity gel overnight, and the western blot was used to detect whether the complex of PZP and GRP78 was formed using indicated antibodies.

To further confirm the direct binding between cell surface GRP78 and recombinant PZP protein, excess MBP‐PZP‐His recombinant protein was reattached to the HisTrap FF affinity beads with RIPA buffer in a 1.5 mL tube. Previously isolated plasma membrane proteins derived from fasted or refed BAT were incubated with beads overnight at 4 °C. After washed for three times with PBS buffer, the beads were re‐suspended with SDS loading buffer, boiled for 15 min and the supernatant after centrifuging were analyzed by western blot.

### Recombinant Adeno‐Associated Viral System for Liver Specific Deletion of PZP

For liver‐specific deletion of PZP, the recombinant adeno‐associated viral (rAAV) system was used. In our rAAV system, the expression of Cas9 was driven by a mouse TBG promoter. This cassette was packaged into a single‐stranded rAAV 2/8 vector (Addgene, pX602). 9 SgRNAs sequence targeting mouse PZP exons designed by ZhangFeng lab website was driven by a U6 promoter. These constructs were packaged into an AAV2/8 vector. One SgRNA (Sense: CACCGTAACTTCCGTCGTGTCTCCAC, antisense: AAACGTGGAGACACGACGGAAGTTAC) targeting AACTTCCGTCG‐ TGTCTCCACTTGGGT in PZP exon 11 was chosen in the following animal experiments according to the editing efficiency test results in vitro as described by Ran F.A. et, al.^[^ ^45^ ^]^

For AAV virus production, 7 ug AAV shuttle vector, 20 ug Delta F6 helper plasmids and 7 ug RC2/8 plasmids were co‐transfected into 80–90% confluent 293FT cell on 15 cm dish by mean of fourfold PEI delivery agent. The medium was changed 16 h after transfection using complete media, and cells were cultured for 48 h. Then viruses were harvested through scraping cells into a 50 mL tube and centrifuging at 1000 rpm for 10 min. Cells were freeze‐thawed three times between lipid nitrogen and water bath to release the viruses, then the viruses were purified by density gradient centrifugation using an opti‐prep agent. QPCR determined titer of virus, serial concentration of AAV vector were set as standard substance.

3‐week‐old mice were injected with control and PZP specific SgRNA virus at 2^ 10^11^ genome copies by tail vein injection. 2 weeks later, the mice were euthanized, QPCR and western blotting were used to detect the knockout efficiency.

### Statistical Analysis

For in vivo experiment, 7–10 mice were randomly assigned to each group to reduce the individual differences. For in vitro experiment, a minimum of 3 replicates were designed, where n values represent independent biological replicates for isolated individual mice for in vivo experiments, number of test repeats and cell experiments in vitro. Specific details of the n value are noted in each figure legend. Statistical analyses were performed with GraphPad Prism version 9.0. Outliers were evaluated using the ROUT method (*Q* = 1%).^[^ ^76^ ^]^ Data were expressed as mean ± SEM. Unpaired two‐tailed Student's *t*‐test was used for two‐group comparison, one‐way ANOVA and two‐way ANOVA with Tukey's post‐test were used for multiple comparisons involving two independent variables, *p*‐values were calculated to determine statistical differences. ***p < 0.001, ** *p*  < 0.01, and * *p*  < 0.05 were considered to be significant.

## Conflict of Interest

The authors declare no conflict of interest.

## Authors’ Contributions

J.L. and X.X.J. contributed equally to this work. W.Z.J. and Z.Z.Y designed experiments, analyzed data, and revised the manuscript. J.L. designed and performed experiments, analyzed data, and wrote the manuscript. X.X.J. performed experiments, analyzed data, and wrote the manuscript. M.D., X.M.L., Y.Y.H., H.L.Z., R.C.Y., H.Q.Z., and C.L.Y. helped with the experiments. Q.W.S. and Z.Y.Z. performed experiments of human samples. L.C. and Y.F.W. revised the manuscript.

## Supporting information

Supporting Information

Supplemental Table 1

## Data Availability

The data that support the findings of this study are available from the corresponding author upon reasonable request.
