# Supplementary material for: Hepatokine Pregnancy Zone Protein Governs the Diet‐Induced Thermogenesis Through Activating Brown Adipose Tissue
Source: Adv Sci (Weinh). 2021 Sep 13;8(21):2101991. doi: 10.1002/advs.202101991 (PMC8564441; doi:10.1002/advs.202101991)
Supplement: Supplementary file 1 — Supporting Information [file ADVS-8-2101991-s001.pdf]

© 2021 Wiley-VCH GmbH

ADVANCED SCIENCE
Open Access

Supporting Information

for *Adv. Sci.,* DOI: 10.1002/advs.202101991

Hepatokine pregnancy zone protein governs the diet-induced thermogenesis through activating brown adipose tissue

Jun Lin, Xiaoxiao Jiang, Meng Dong, Xiaomeng Liu, Qiwei Shen, Yuanyuan Huang, Hanlin Zhang, Rongcai Ye, Huiqiao Zhou, Chunlong Yan, Shouli Yuan, Xiangnan Wu, Li Chen, Yanfang Wang, Min He, Yi Tao, Zhaoyun Zhang, and Wanzhu Jin**

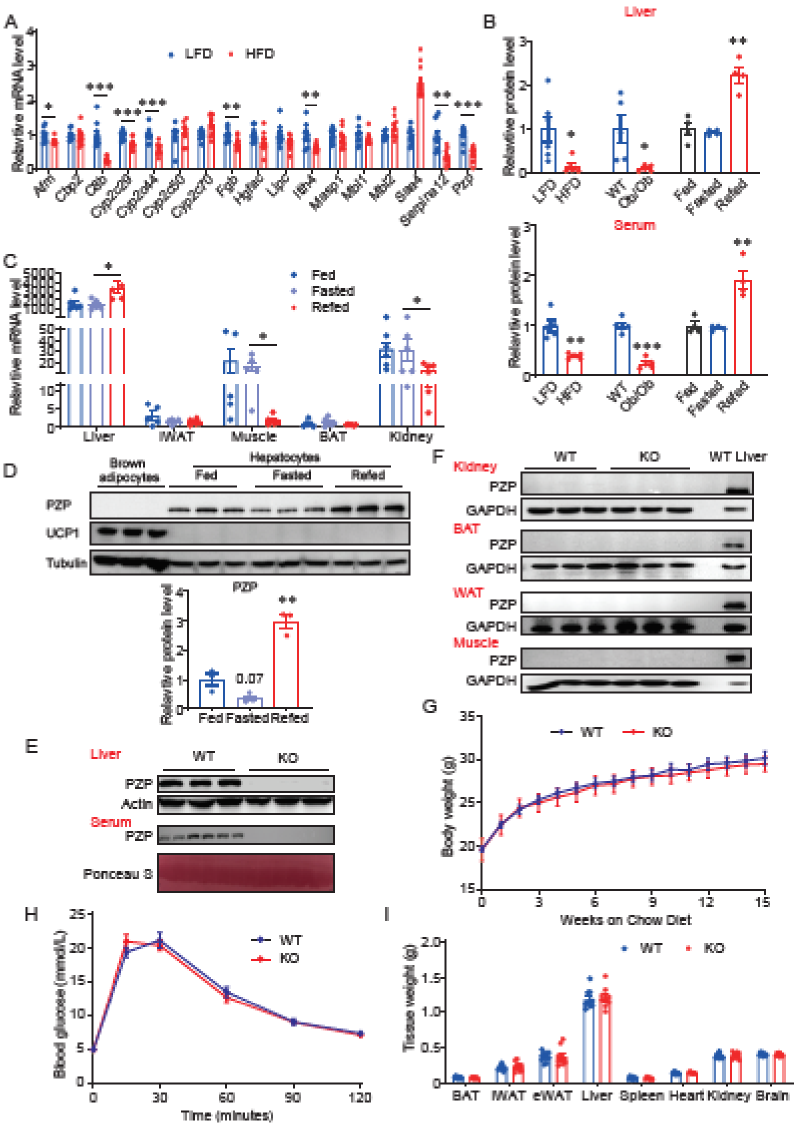

Lin et al. Supplementary Fig. 1

Supplementary Fig 1. PZP Null Mice Appears Normal on Chow Diet. Related to Figure 2

(A) Relative mRNA expression of candidates in liver from C57B6 mice on LFD or HFD (n = 9-10).

(B) Quantification of PZP protein level of figure 1F and 1G (n = 4-5).

(C) Relative mRNA expression of PZP in liver, iWAT, muscle, BAT and Kidney from fed or fasted (24 hrs) or refed (6 hrs) mice (n = 5-6).

(D) Immunoblots of brown adipocytes and primary hepatocytes which were cultured in feeding, fasting or refeeding medium using indicated antibodies.

(E) Immunoblots of total liver lysates and serum sample from WT and PZP KO mice using indicated antibodies.

(F) Immunoblots of kidney, BAT, WAT, and muscle lysates from WT and PZP KO mice using indicated antibodies.

(G-I) Body weight (G), GTT (H) and tissue weight (I) of WT and PZP KO mice are shown (n = 8-11).

Data are shown as mean ± SEM. Two-tailed Student's t test (**A**) or One-way ANOVA (**B, C** and **D**) with multiple comparisons and Tukey's post-test were performed; ***P<0.001, **P < 0.01 and *P < 0.05 were considered to be significant.

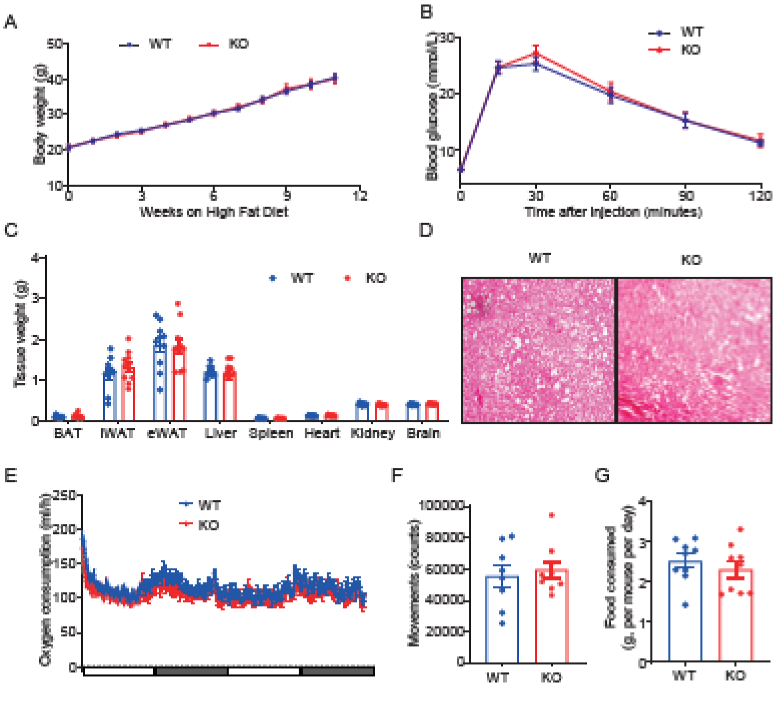

Lin et al. Supplementary Fig. 2

Supplementary Fig 2. PZP Null Mice Appears Normal upon HFD. Related to Figure 2

WT and PZP KO Mice chronically subjected to HFD for 12 weeks.

(A-C) Body weight (A), GTT (B) and tissue weight (C) of WT and PZP KO mice are shown (n = 10).

(D) Representative H&E staining images of liver from WT and PZP KO mice (scale bar = 100 µm).

(E-G) Oxygen consumption (E), physical activity (F) and food consumption (G) of WT and PZP KO mice are shown (n = 9).

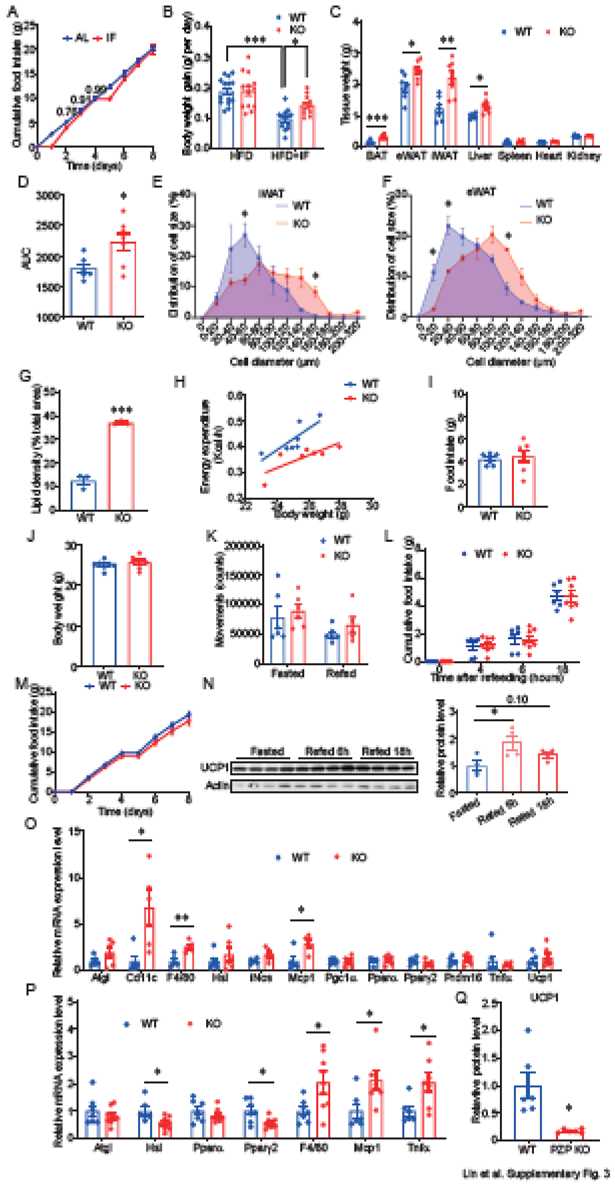

Supplementary Fig 3. Metabolic Characterization of PZP Null mice upon HFD and IF. Related to Figure 2

(A) Food consumption of AL group and IF group mice (n = 5). The percentage ratio of IF group to AL group were labeled in the lines.

(B) Average body weight gain of WT and PZP KO mice fed on HFD or HFD plus IF regimen (n = 11-13).

(C) Tissue weight of WT and PZP KO mice (n = 7-8).

(D) Area under the curve (AUC) of GTT of WT and PZP KO mice is shown (n = 7-8).

(E-F) Quantification of cell size of iWAT (E) and eWAT (F) from WT and PZP KO mice upon HFD and IF (n= 3).

(G) Quantification of lipid density of liver from WT and PZP KO mice upon HFD and IF (n= 3).

(H) Relation between average EE and body weight of WT and PZP KO mice which were used to oxygen consumption experiment in Figure 2F (n = 7).

(I-J) Food intake (I) and body weight (J) of WT and PZP KO mice which were used to oxygen consumption experiment in Figure 2F (n = 7).

(K-M) Physical activity (K), food intake during refeeding (L) and cumulative food intake (M) of WT and PZP KO mice (n = 6-9).

(N) Immunoblots of total BAT lysates from fasted (24 hrs), refed (6 hrs and 18 hrs) mice using indicated antibodies.

(O-P) Relative mRNA expression of adipogenic, thermogenic and inflammatory genes in iWAT (O) and eWAT (P) from WT and PZP KO mice (n = 5-8).

(Q) Quantitative result of immunoblots from Figure 2K (n = 6).

Data are shown as mean ± SEM. Two-tailed Student's t test (**C, D, E, F, G, O, P** and **Q**) or One-way (**H**, **N**) or two-way ANOVA (**B**) with multiple comparisons and Tukey's post-test were performed; ***P<0.001, **P < 0.01 and *P < 0.05 were considered to be significant.

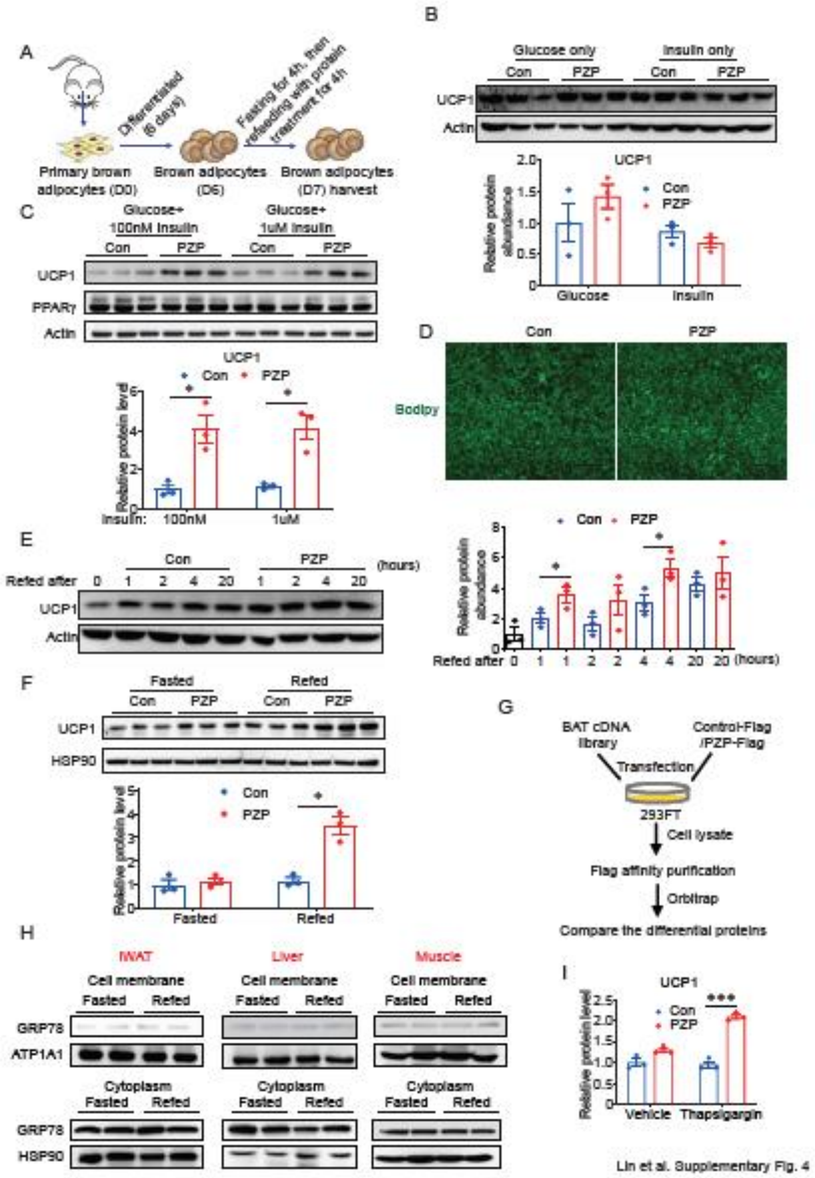

Lin et al. Supplementary Fig. 4

Supplementary Fig 4. PZP Cell-autonomously Increases UCP1 Expression in Brown Adipocytes. Related to Figure 3

(A) Schematic shows the procedure of in-vitro cell culture experiments.

(B-C) Immunoblots of cell lysate from differentiated BA which were cultured in different refeeding medium (B and C) supplemented with 100 ng/mL PZP protein using indicated antibodies.

(D) Representative microscopic images of differentiated brown adipocytes (BA) after protein treatment.

(E) Immunoblots of cell lysate from differentiated BA during the time course of refeeding supplemented with 100 ng/mL PZP protein.

(F) Immunoblots of cell lysate from differentiated BA cultured in feeding, fasting or refeeding medium supplemented with 100 ng/mL PZP protein.

(G) Schematic shows the experimental procedure of screening receptor of PZP.

(H) Immunoblots of cell membrane and cytoplasmic fractions from iWAT, liver and muscle using indicated antibodies.

(I) Quantitative result of immunoblots from Figure 3K (n= 3).

Data are shown as mean ± SEM. Two-way ANOVA (**B, C**, **E, F** and **I**) with multiple comparisons and Tukey's post-test were performed; $^{**}P < 0.01$ and $^{*}P < 0.05$ were considered to be significant.

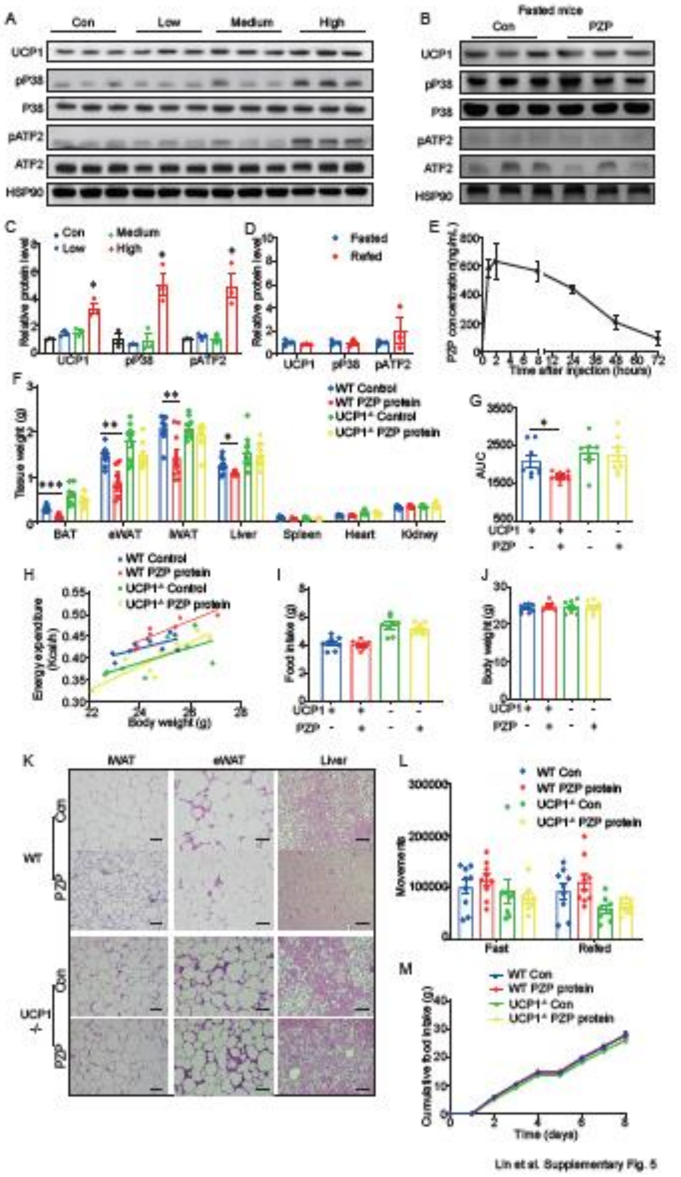

Supplementary Fig 5. Increased Circulating PZP Promotes UCP1 Level in C57B6 Mice. Related to Figure 5

We performed hypodermic injection of PZP protein when refeeding began.

(A) Immunoblots of total BAT lysates from control, low dose (40 μg/kg) medium dose (200 μg/kg) and high dose (1mg/kg) group mice using indicated antibodies. These mice were treated with one single injection of PZP protein.

(B) Immunoblots of total BAT lysates from fasted mice with or without PZP protein injection (1mg/kg) using indicated antibodies.

(C-D) Quantitative result of immunoblots from supplementary figure 5A (C) and supplementary figure 5B (D) (n= 3).

(E) Serum PZP level of WT mice in indicated timepoints after single injection of PZP protein (1mg/kg).

(F-G) Tissue weight (F) and AUC of GTT (G) of WT and UCP1-/- mice treated with or without PZP protein (1 mg/kg) are shown (n = 8-10).

(H) Relation between average EE and body weight of WT and UCP1-/- mice treated with or without PZP protein (1 mg/kg) which were used to oxygen consumption experiment in Figure 5D (n = 7).

(I-J) Food intake (I) and body weight (J) of WT and UCP1-/- mice treated with or without PZP protein (1 mg/kg) which were used to oxygen consumption experiment in Figure 5D (n = 7).

(K) Representative H&E staining images of iWAT, eWAT and liver from WT and UCP1-/- mice treated with or without PZP protein (scale bar = 100 μm).

(L-M) Physical activity (L) and cumulative food intake (M) of WT and UCP1 -/- mice (n = 8-9).

Data are shown as mean ± SEM. One-way ANOVA (**C**, **F** and **G**) with multiple comparisons and Tukey's post-test were performed; ***P < 0.001, **P < 0.01 and *P < 0.05 were considered to be significant.

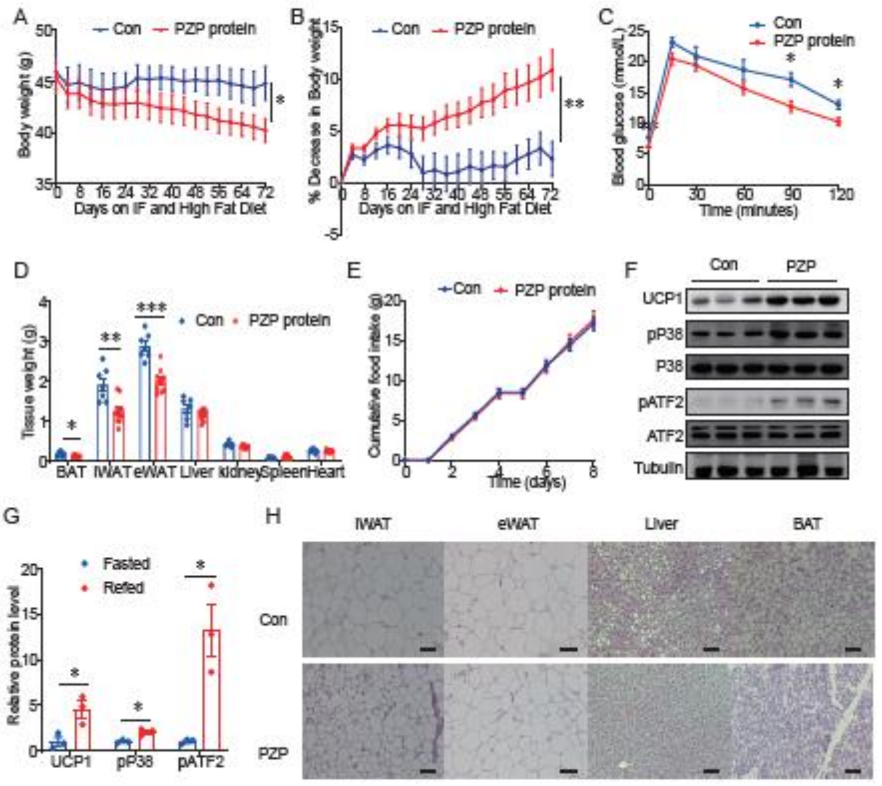

Lin et al. Supplementary Fig. 6

Supplementary Fig 6. Circulating PZP Rescues the established DIO in PZP KO Mice.

We performed hypodermic injection of PZP protein when refeeding began.

(A-E) Body weight (A), percent decrease (%) in body weight (B), GTT (C) tissue weight (D) and cumulative food intake (E) of PZP KO mice treated with or without PZP protein (1 mg/kg) are shown (n = 8-10).

(F-G) Immunoblots (F) and quantitative result (G) of total BAT lysates from PZP KO mice treated with or without PZP protein using indicated antibodies (n = 3).

(H) Representative H&E staining images of BAT, iWAT, eWAT and liver from PZP KO mice with or without PZP protein (scale bar = 100 μm).

Data are shown as mean ± SEM. Two-way repeated measures ANOVA (**A, B, C, D** and **G**) with multiple comparisons and Tukey's post-test were performed; **P < 0.01 and *P < 0.05 were considered to be significant.

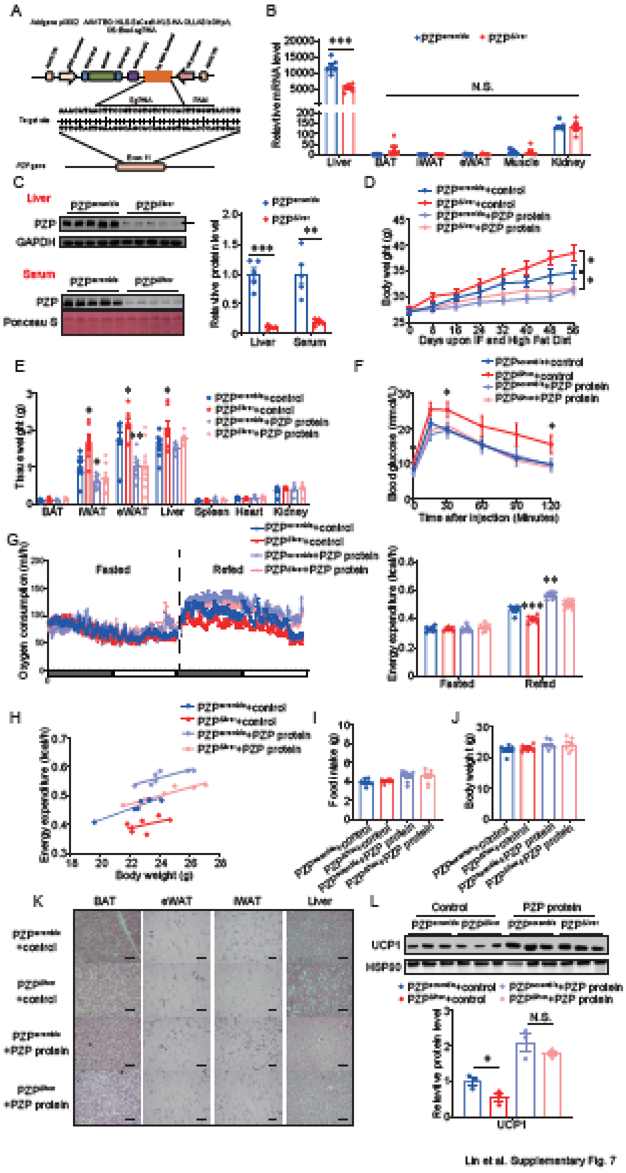

Supplementary Fig 7. Anti-obesity Effect of IF is Impaired in PZPΔliver Mice But Rescued by PZP Protein.

(A) AAV-TBG-SaCas9 targeting murine *Pzp*. Schematic representation of AAV vector, guide RNA sequence, and *Pzp*-Exon11 target site. ITR inverted terminal repeats, Myc c-myc, NLS nuclear localization signal sequence, bGH-poly (A) bovine growth hormone polyadenylation signal, PAM protospacer adjacent motif.

(B) Relative mRNA expression of Pzp in liver, BAT, iWAT, eWAT, muscle and kidney from $PZP^{scramble}$ and $PZP^{\Delta liver}$ mice (n = 7-8).

(C) Western blot analysis of PZP protein level in liver and serum from $PZP^{scramble}$ and $PZP^{\Delta liver}$ mice (n = 5).

(D-F) Body weight (D), tissue weight (E) and GTT (F) of $PZP^{scramble}$ and $PZP^{\Delta liver}$ mice treated with or without PZP protein (1 mg/kg) are shown (n = 7-8).

(G) Oxygen consumption rate (left panel) and energy expenditure (right panel) of $PZP^{scramble}$ and $PZP^{\Delta liver}$ mice treated with or without PZP protein (1 mg/kg) (n = 7). These experiments were performed when mice were upon HFD and IF after 2 weeks without difference in body weight between groups, which showed PZP deficiency in liver caused a marked decrease in whole-body energy expenditure and PZP protein injection dramatically increased the oxygen consumption and energy expenditure of $PZP^{scramble}$ and $PZP^{\Delta liver}$ mice.

(H) Relation between average EE and body weight of $PZP^{scramble}$ and $PZP^{\Delta liver}$ mice treated with or without PZP protein (1 mg/kg) which were used to oxygen consumption experiment in Figure S7G (n = 7).

(I-J) Food intake (I) and body weight (J) of $PZP^{scramble}$ and $PZP^{\Delta liver}$ mice treated with or without PZP protein (1 mg/kg) which were used to oxygen consumption experiment in Figure S7G (n = 7).

(K) Representative H&E staining images of BAT, iWAT, eWAT and liver from $PZP^{scramble}$ and $PZP^{\Delta liver}$ mice with or without PZP protein (scale bar = 100 μm).

(L) Immunoblots and quantitative results of total BAT lysates from $PZP^{scramble}$ and $PZP^{\Delta liver}$ treated with or without PZP protein using indicated antibodies (n = 3).

Data are shown as mean ± SEM. Two-tailed Student's t test (**B** and **C**) or one-way (**G**) or two-way repeated measures ANOVA (**D, E, F** and **L**) with multiple comparisons and Tukey's post-test were performed; ***$P < 0.001$, **$P < 0.01$ and *$P < 0.05$ were considered to be significant.
