# Supplementary material for: Hepatokine Pregnancy Zone Protein Governs the Diet‐Induced Thermogenesis Through Activating Brown Adipose Tissue
Source: Adv Sci (Weinh). 2021 Sep 13;8(21):2101991. doi: 10.1002/advs.202101991 (PMC8564441; doi:10.1002/advs.202101991)
Supplement: Supplementary file 2 — Supplemental Table 1 [file ADVS-8-2101991-s002.pdf]

Supplementary table 1 Primers for RT-PCR

Gene	Forward primer	Reverse primer
Serpina12	AATGTGTATGATGCGGACA	TACTGCCACCTGCCTCGAA
Mbl2	AGGAAAACTCGGCCATCCAG	TGTTGGGCTCCCCATCATTC
C8b	CCCACAGCAAAGCAAGTTCC	CGTGTACTCGTAGATGCCCC
Cyp2c29	ATGACCTCTTTGGTGCAGGG	TAGTGGGGAGGAGGTCGATG
Cbp2	CTCACAAGAACAACCGCTGC	TCACCTCTGGCTCAGACTCA
Cyp2c44	GTGAAGGAGCACCAGGAGTC	TTGGCTTGCACCTCTGGATG
Afm	CTTTGAGCATCTGAAAGCTGAT	TTGTGAACAAGCACAGCCAC
Cyp2c50	CCAATGGCTCACCCTGTGAT	CCGTTTTCCTGCAACCAAGG
Mbl1	GAGAACCAGGTCAAGGGCTC	TGCCAGCTTCTCCTCAATGG
Masp1	CCGGAGGGGTTTCGAATCAA	GGTCTGCTCAGTATCGGTGG
Lipc	GCAGCTTCAGCCAAGGTCTA	CTTTTGTTCCCAGCAGCGAC
Cyp2c70	TTGTGGCTTTCTCAGCAGGA	AACTGGCTTGGTGTCGATGT
Hgfac	CCACGGATGTGACACAGACA	ACATTTTCATCCATGTGGCCC
Fgb	ACGATGAACCGACGGATAGC	TCTGGTTGGCAGTAGCCTTG
Saa4	AGAGCCTATCGGGACAACCT	CATTCCTCGGCTTTCTGGGT
Itih4	GCCCTCCGTTGCACAATATC	AGACCCTGACTGGGGTCTAC
Acadm	ACTCGAAAGCGGCTCACAA	ACGGGGATAATCTCCTCTCTGG
Atgl	ATATCCCACTTTAGCTCCAAGG	CAAGTTGTCTGAAATGCCGC
Cd11c	TTCAAGGAGACAAAGACCCG	AGAGAAAAGTTGAGGCGAAGAG
Cd36	GCGACATGATTAATGGCACAG	GATCCGAACACAGCGTAGATAG
Cebpb	TGACGCAACACACGTGTAACTG	AACAACCCCGCAGGAACAT
Cpt1a	AGACAAGAACCCCAACATCC	CAAAGGTGTCAAATGGGAAGG
Cyclophilina	CAAATGCTGGACCAAACACAA	GCCATCCAGCCATTCAGTCT
F4/80	CTTTGGCTATGGGCTTCCAGTC	GCAAGGAGGACAGAGTTTATCGTG
Hsl	CTGAGATTGAGGTGCTGTCG	CAAGGGAGGTGAGATGGTAAC
iNos	GCAAACATCACATTCAGATCCC	TCAGCCTCATGGTAAACACG
Mcp1	GGAGAGCTACAAGAGGATCAC	TGATCTCATTTGGTTCCGATCC
Pgc1a	ACAGCTTTCTGGGTGGATTG	TGAGGACCGCTAGCAAGTTT
Ppara	AGCCTCAGCCAAGTTGAAGT	TGGGGAGAGAGGACAGATGG
Pparg2	TCGCTGCTGCACTGCCTCTG	GAGAGGTCCACAGAGCTGATT
Pzp	ACCCATATACAAACCAGGACAG	CTGCCAGATGGATATTTTGCC
Prdm16	GAAGTCACAGGAGGACACGG	CTCGCTCCTCAACACACCTC
Ucp1	GGCAAAAACAGAAGGATTGC	TAAGCCGGCTGAGATCTTGT
Tnfa	TGGGCCTCTCATGCACCACC	GAGGCAACCTGACCACTCTCCCT

Table S2 Blood parameters of animals

	Insulin (μIU/ml)	Free fatty acid (mM)	Cholesterol (mg/dL)	Triglyceride (mg/dL)
PZP WT	17.68±1.20	0.25±0.02	140.38±3.91	80.45±1.39
PZP KO	19.79±1.01	0.32±0.01 *	153.27±3.26 *	87.67±2.49 *
WT control	16.53±0.90	0.21±0.02	142.5±2.91	99.11±3.1
WT PZP protein	13.24±0.63**	0.15±0.02	129.38±2.65 *	84.44±1.94 **
UCP1-/- control	16.18±0.91	0.17±0.01	169.8±5.73	65.8±2.19
UCP1-/- PZP protein	16±0.94	0.30±0.05	170.89±5.14	68.11±3.44
PZP KO +control	11.28±0.82	0.30±0.01	162.33±5.67	120.17±8.64
PZP KO +PZP protein	10.55±0.94	0.23±0.01 *	139.29±3.62 *	127.57±3.72

one-way ANOVA with multiple comparisons and Tukey's post-test were performed; **P < 0.01 and *P < 0.05 were considered to be significant.

Table S3 clinical parameters of BMI figure

ID	PZP concerntration (ug/ml)	sex	age	height (cm)	weight (kg)	BMI
F0003	5.17	female	68	150	80	35.56
F0007	1.52	female	69	161	80	30.86
F0011	5.19	female	54	168	95	33.66
F0012	5.8	female	49	172	120	40.56
F0013	2.16	male	30	182	132	39.85
F0015	1.83	female	36	165	83	30.49
F0016	3.18	female	22	168	128	45.35
F0018	2.75	female	18	170	183	63.32
F0019	2	female	16	160	105	41.02
F0020	2.17	male	65	175	108	35.27
F0022	2.56	male		185	128	37.4
F0024	1.86	female	49	165	80	29.38
F0025	2.88	female	27	164	85	31.6
F0026	1.93	female	15	176	128	41.32
F0029	2.07	male	24	178	115	36.3
F0030	3.24	female	24	168	111	39.33
F0031	2.05	female	49	158	75	30.04
F0035	2.11	female	58	159	110	43.51
H0010	2.49	female	51	160	65	25.39
H0017	1.81	male	45	176	76	24.54
H0020	1.97	male	51	170	77	26.64
H0033	5.12	male	51	170	75	25.95
H0034	3.58	male	34	178	80	25.25
H0037	3.03	male	48	170	70	24.22
H0040	3.05	male	32	175	75	24.49
L0001	3.27	male	61	175	73	23.84
L0003	8.49	female	66	165	64	23.51
L0026	3.03	male	74	164	58	21.56
L0030	1.62	female	54	163	63	23.71
L0032	9.35	female	35	164	55	20.45
L0037	4.49	female	49	168	68.8	24.38
L0039	1.46	female	49	150	55	24.44
L0040	3.05	female	57	161	61	23.53
L0042	18.21	female	46	168	57	20.2
L0043	4.56	female	41	162	46	17.53
L0045	4.06	female	56	160	59	23.05
L0046	11.2	female	39	155	57	23.73
L0047	7.77	female	36	160	57.5	22.46
L0048	2.23	male	53	172	69	23.32
L0049	8.92	female	35	168	64	22.68
L0050	2.82	male	52	170	65	22.49
L0051	2.94	female	51	162	56	21.34
L0054	3.04	male	44	172	65	21.97
L0055	4.12	female	27	155	50	20.81

Table S4 clinical parameters of OGTT figure

	PZP concerntration (ug/ml)						
ID	OGTT 0h	OGTT 2h	sex	age	height (cm)	weight (kg)	BMI
1	2.07	4.63	male	62	173	65	21.7
2	1.85	2.97	male	65	179	90	28.1
3	1.96	3.99	male	32	170	85	29.4
4	13.13	15.53	female	29	173	63	21
5	1.53	2.26	male	31	184	90	26.6
6	1.54	1.76	female	44	165	70	25.7
7	2.17	8.33	female	49	160	62	24.2
8	1.66	3.21	male	28	169	80	28
9	3.51	2.73	male	68	182	75	22.6
10	3.62	4.09	female	23	170	63	21.8
11	3.18	3.24	female	56	158	55	22
12	7.85	8.12	male	37	175	65	21.2
13	2.96	3.82	male	69	173	73	24.4
14	3.28	3.38	male	72	169	74	25.9
15	1.89	2.22	female	54	155	62.5	26
16	2.44	2.33	female	60	158	58	23.2
17	1.47	6.48	female	35	158	68	27.2
18	1.67	4.56	male	32	175	90	29.4
19	29.65	33.20	female	25	161	51	19.7
20	2.46	13.47	female	56	161	60	23.1
21	2.05	3.66	female	35	161	54	20.8
22	4.60	4.59	male	33	183	78	23.3
23	8.61	20.31	male	52	182	70	21.1
24	2.07	1.98	male	55	168	76	26.9
